# Transition-metal-catalyzed synthesis of quinazolines: A review

**DOI:** 10.3389/fchem.2023.1140562

**Published:** 2023-03-16

**Authors:** Rekha Tamatam, Seok-Ho Kim, Dongyun Shin

**Affiliations:** ^1^ College of Pharmacy, Gachon University, Incheon, Republic of Korea; ^2^ Gachon Pharmaceutical Research Institute, Gachon University, Incheon, Republic of Korea; ^3^ College of Pharmacy, Kangwon National University, Chuncheon, Gangwon-do, Republic of Korea

**Keywords:** quinazoline, transition-metal, catalysis, synthesis, mechanism

## Abstract

Quinazolines are a class of nitrogen-containing heterocyclic compounds with broad-spectrum of pharmacological activities. Transition-metal-catalyzed reactions have emerged as reliable and indispensable tools for the synthesis of pharmaceuticals. These reactions provide new entries into pharmaceutical ingredients of continuously increasing complexity, and catalysis with these metals has streamlined the synthesis of several marketed drugs. The last few decades have witnessed a tremendous outburst of transition-metal-catalyzed reactions for the construction of quinazoline scaffolds. In this review, the progress achieved in the synthesis of quinazolines under transition metal-catalyzed conditions are summarized and reports from 2010 to date are covered. This is presented along with the mechanistic insights of each representative methodology. The advantages, limitations, and future perspectives of synthesis of quinazolines through such reactions are also discussed.

## 1 Introduction

**FIGURE 1:**
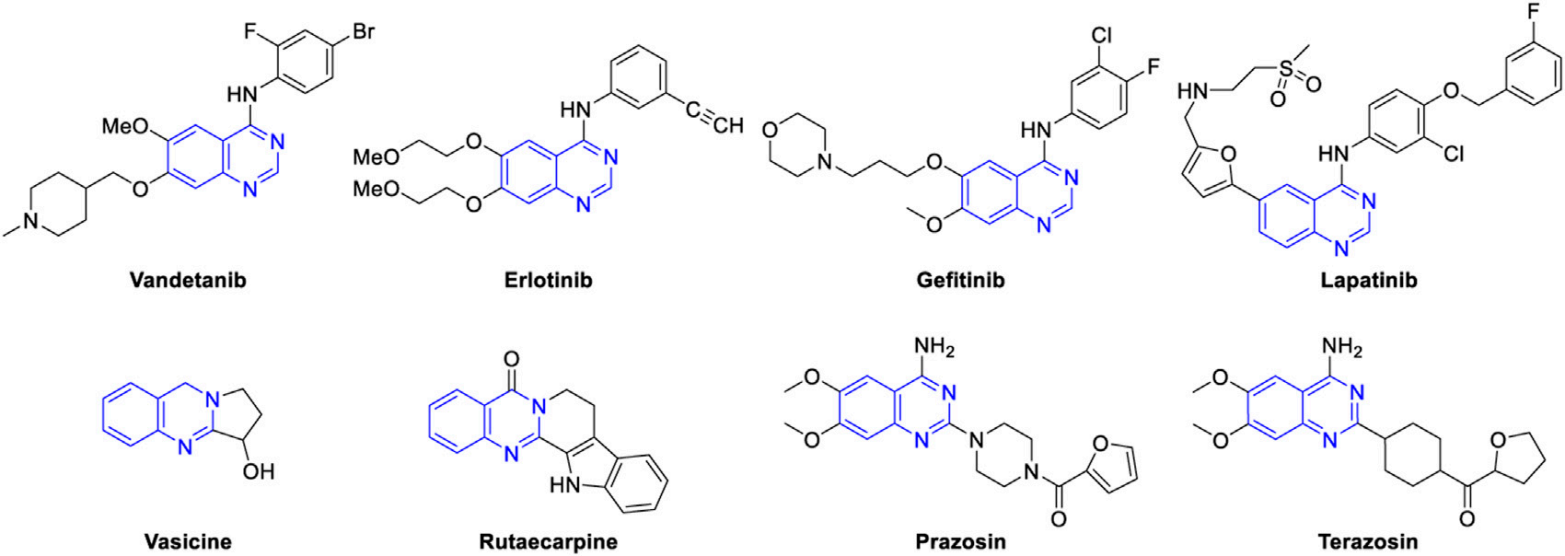
Quinazoline-containing drugs and natural products.

Nitrogen-containing heteroaromatic compounds are important components of pharmaceuticals, agrochemicals, dyes, and other functional materials (Michael, 2008). Quinazolines comprise a family of nitrogen-based bicyclic heteroarenes that are considered attractive targets by medicinal chemists as these motifs are the constituents of bioactive molecules that show antibacterial (Jafari et al., 2016), antiviral (Wan et al., 2015), anti-inflammatory (Chandrikaa et al., 2008), anticancer (Shagufta, 2017), anticoccidial (Ye et al., 2010), antimutagenic (Cakici et al., 2010), antileishmanial (Van Horn et al., 2014), antimalarial (Bule et al., 2017), antioxidant (Gilson et al., 2017), antiobesity (Sasmal et al., 2012), anti-tubercular (Kunes et al., 2000), neuroprotective (Zhang et al., 2018a), and hypertensive activities (Rahman et al., 2014). In fact, Vandetanib is the first drug approved by FDA for the treatment of medullar thyroid cancer (Chau and Haddad, 2013). Several antitumor drugs, e.g., erlotinib (Higgins et al., 2004) and gefitinib (Sordella et al., 2004) are currently used in the market. Lapatinib is an effective inhibitor for epidermal growth factor and is used in combination therapy for breast cancer (Johnston et al., 2009). Vasicine, a natural quinazoline alkaloid isolated from *Adhatoda vasica,* displays bronchodilatory activity both *in vitro* and *in vivo* (Gupta et al., 1977). Rutaecarpine (Tian et al., 2019) is another alkaloid which has been used to treat gastrointestinal disorders, inflammation, headache, and postpartum hemorrhage, and recently, 2-arylquinazolines were identified as novel inhibitors of HIV-1 capsid assembly (Machara et al., 2016). Prazosin (Rosini et al., 2003) is utilized to treat high blood pressure, anxiety, and panic disorder. Terazosin is another important drug on the market for the treatment of symptomatic benign prostatic hyperplasia (Figure 1) (Wilt et al., 2002; Anwarul Islam et al., 2005).

Additionally, the unique photochromic behavior of quinazoline scaffolds has been exploited to elucidate cellular signaling processes of epidermal growth factor receptor (Liu et al., 2018) (EFGR) and α_1_-adrenergic receptors (Ma et al., 2016). Furthermore, quinazolines have been identified as light-emitting materials for electronic devices (Lipunova et al., 2018). Moreover, quinazolines also serve as valuable intermediates for preparation of functionalized materials (Zhao et al., 2013). More than 70% of the proprietary drugs are composed of *N*-heterocycles, as reported in a survey (Based on annual sales of drugs in the year 2009, 2009). Owing to their wide application in the pharmaceutical field, several synthetic methods have been developed over the past few decades. Griess in 1869 reported the first example of the synthesis of quinazolines from cyanogen and anthranilic acid (Griess, 1869). Nevertheless, these classical and general approaches are mostly associated with harsh reaction conditions, limited substrate generality and availability of starting materials, multistep reaction sequences, requirement of pre-functionalized substrates and excess oxidants, and generation of auxiliary waste. Hence, the development of new and alternative methods that involve inexpensive and readily available starting materials is desirable. The transition-metal-catalyzed formation of *N*-heterocycles continues to be an active area of research because these metals easily lose and gain electrons, and most of them are malleable, ductile, easily available, and act as efficient catalysts. In organic transformations, transition-metal-catalyzed reactions have advantages such as mild reaction conditions and compatibility with an extensive range of functionalities. As a result, transition-metal-catalyzed approaches are deliberated most assiduously by activating the starting materials *via* coordination, ligand exchange/promotion, elimination, etc. The selectivity of these catalysts can be achieved by ligand alteration.

**FIGURE 2:**
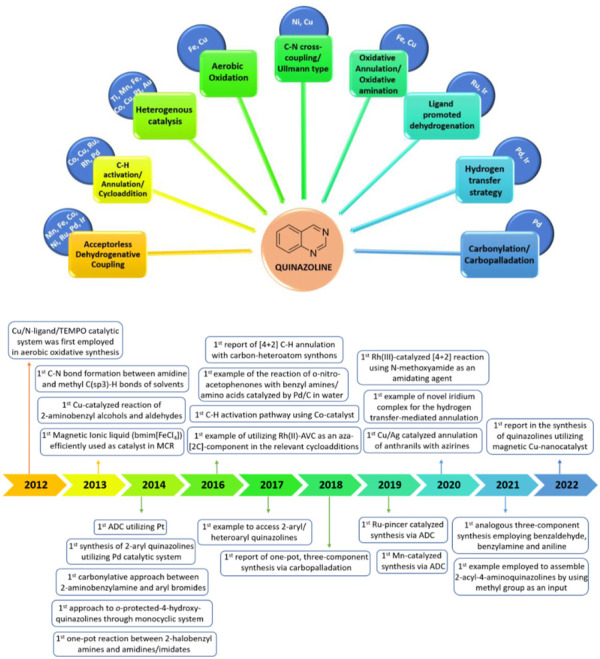
First-, second-, and third-row transition-metal-catalyzed synthesis of quinazolines and first reported examples involved in the synthesis of quinazolines (2012–2022).

In recent years, reviews have been presented on the synthetic approaches and biological activity of quinazolines (Wang and Gao, 2013; Srivastava and Srivastava, 2015; Hameed et al., 2018; Auti et al., 2020; Faisal and Saeed, 2021). However, transition-metal-catalyzed synthesis of quinazolines has not been properly highlighted. After a thorough literature study, the transition-metal-catalyzed synthesis of quinazolines was achieved, as illustrated in Figure 2. This review is divided into three categories: A) first-row, B) second-row, and C) third-row transition-metal catalysts. This review highlights this topic by discussing the advantages, limitations, shortcomings, and mechanistic aspects of representative methods over the last decade (2010–2022) in a chronological manner. This is presented alongside the first reported examples of different catalysts or bond-formation reactions involved in the construction of quinazoline scaffolds (Figure 2).

## 2 First-row transition metal catalysts

### 2.1 Titanium

Titanium is the second most abundant transition metal and the ninth most abundant metal in the Earth’s crust. Ti plays a key role in the synthesis of fine chemicals, agrochemicals, and pharmaceuticals, and industrial processes. Ti catalysts are versatile, less toxic, inexpensive, and biocompatible. However, its use as a catalyst has been overlooked because of its oxophilic Lewis acidity, which renders titanium complexes less tolerant to functional groups than their transition-metal counterparts. Ti complexes facilitate excellent transformations such as reductive coupling (McMurry et al., 1978), radical reactions (Nugent and RajanBabu, 1988), hydroaminoalkylation (Luhning et al., 2017), and hydroamination (Janssen et al., 2010).

**SCHEME 1:**
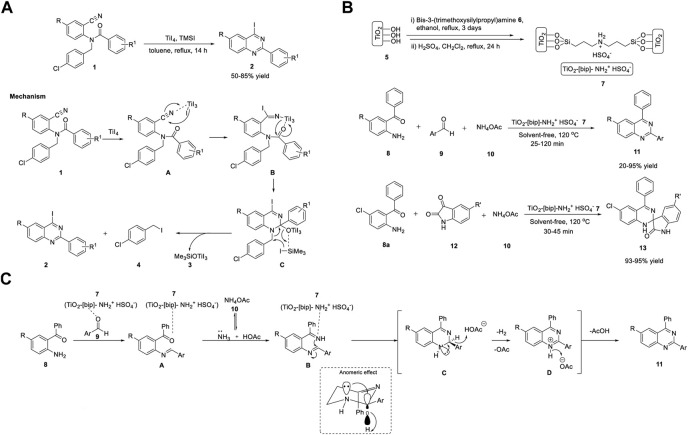
**(A)** Titanium tetraiodide and TMSI synergistically induced synthesis of 2-aryl-4-iodoquinazolines **(B)** Synthesis of quinazolines and spiro-quinazolines using Ti catalyst **(C)** Proposed Mechanism for the construction of quinazolines.

Shimizu et al. (2019) reported that the synthesis of iodoquinazolines **2** using titanium tetraiodide/trimethylsilyl iodide synergistically induced the iodination–cyclization of benzamides **1**. 2-Aryl-4-iodoquinazolines **2** were synthesized from *N*-(4-chlorobenzyl)-*N*-(2-cyanoaryl)benzamides **1** (as a nitrogen source) in the presence of titanium tetraiodide and trimethylsilyl iodide in toluene under reflux conditions for 14 h, with 50%–85% yields (Scheme 1). (Hachiya and Shimizu, 2019) The key highlights of this protocol are as follows: benzamide **1** can be prepared from readily available benzonitrile derivatives and the iodine functionality can be applied for further transformations. The only drawback of this procedure is the formation of 1-chloro-4-(iodomethyl)benzene **4** as a by-product in every case.

The proposed mechanism involves the coordination of titanium tetraiodide with the cyano group of *N*-(4-chlorobenzyl)- *N*-(2-cyanoaryl)benzamide **1** to generate Ti intermediate **A**. Intermediate **B** is formed by the nucleophilic addition of iodide ions to the cyano group of intermediate **A**. Subsequent intramolecular cyclization of intermediate **B** gives titanium alkoxide intermediate **C**, which then undergoes aromatization promoted by TMSI as an additive *via* the elimination of [(trimethylsilyl)oxy]titanium(IV) iodide **3** and 1-chloro-4-(iodomethyl)benzene **4** to afford 2-aryl-4-iodoquinazoline **2** (Scheme 1A). The aromatization step is driven by the synergistic effect of TiI_4_ and TMSI, and elimination is promoted by the increased nucleophilicity of iodide ions, resulting from the coordination of titanium alkoxide with the silyl group.

The combination of solid acids and ionic liquids possesses a wide range of benefits, such as easy handling, separation, and reuse procedures, and minimal usage of ionic liquids in reactions (Wight and Davis, 2002). One such example is nanoporous TiO_2_ containing an ionic liquid bridge (TiO_2_-[bip]-NH_2_^+^ HSO_4_^−^). Shirini *et al.* (2020) reported the Ti-catalyzed synthesis of quinazolines **11** and spiroquinazolines **13** *via* the reaction of *o*-aminobenzophenone **8**, benzaldehyde **9**, or isatin **12** and ammonium acetate **10** in the presence of catalyst **7** (TiO_2_-[bip]- NH_2_^+^ HSO_4_^−^) at 120°C under solvent-free conditions (Scheme 1B). (Mazloumi and Shirini, 2020) Catalyst **7** was prepared in two steps, starting from TiO_2_ **5** and bis-3-(trimethoxysilylpropyl)amine **6** in ethanol (Scheme 1B).

The attractive features of this quinazoline synthesis procedure are high reaction rates, short reaction times, easy preparation and handling of catalysts, green experimental protocols, and inexpensive, reusable catalysts. However, electron-withdrawing and electron-donating groups such as -NO_2_, -OH, and -Cl in the *ortho* position of the aromatic aldehyde resulted in lower yields compared to *para*-substituted aldehydes. This is due to the steric hindrance effect, which constitutes a limitation in the scope of the substrate and requires further development.

Mechanistically, a Brønsted acidic titanium catalyst **7** (TiO_2_-[bip]-NH_2_^+^ HSO_4_^−^) activated the carbonyl group, and the reaction occurred in two steps. In the first step, the nucleophilic attack of *o*-aminobenzophenone **8** on the carbonyl carbon of aldehyde **9** and ammonia from ammonium acetate **10** attacks the carbonyl groups of *o*-aminobenzophenone **8** to generate intermediate **A**. In the second step, the ring closure and aromatization of intermediate **A** afforded quinazoline **11** *via* anomeric-based oxidation (Scheme 1C).

### 2.2 Manganese

Manganese is the third most abundant transition-metal in the Earth’s crust. Mn is unique as it is non-toxic and environmentally benign among the 3d-transition-metals. The presence of various catalytically active Mn complexes is due to the variable oxidation states of Mn. Mn catalysts are used in several organic transformations such as C-H activation (Liu and Ackermann, 2016), hydrosilylation (Yang and Wang, 2018), acceptorless dehydrogenative coupling (ADC) (Waiba and Maji, 2020), and hydrogen autotransfer (HA).

Heterogeneous catalysis has emerged as an important approach in cascade organic synthesis (Szollosi et al., 2015). Its advantages include easy catalyst separation, recyclability, and ligand-free conditions (Zhang et al., 2014a). Heterogenous catalysts are employed in the form of metal oxides, metals, and zeolites. Manganese oxide is one such example that is mostly used as a stoichiometric oxidant and rarely as an efficient catalyst (Pal et al., 2013).

**SCHEME 2:**
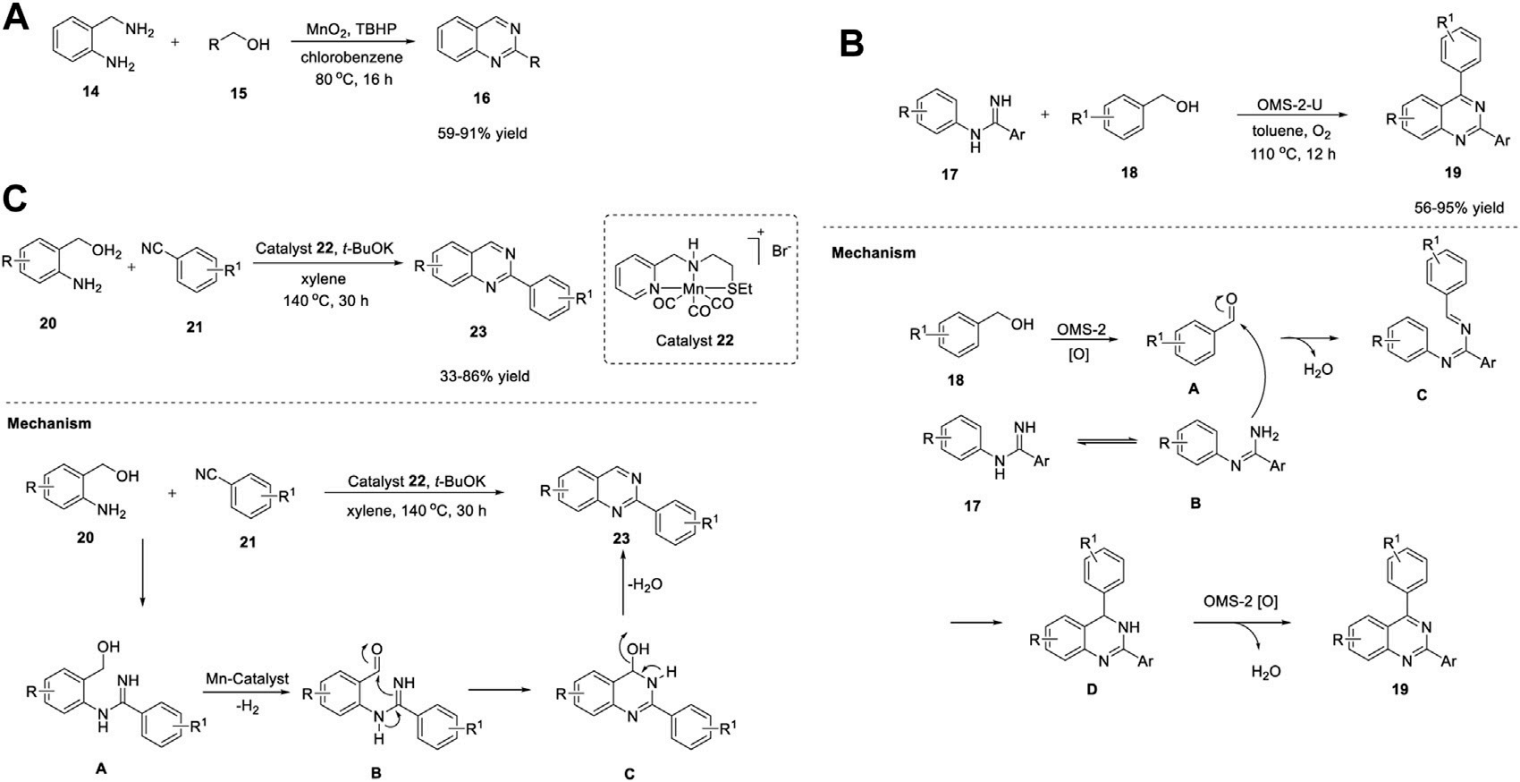
**(A)** Synthesis of quinazolines using MnO_2_ as catalyst **(B)** Synthesis of quinazolines using Manganese oxide OMS-2 catalyst **(C)** Synthesis of quinazolines using Manganese-Pincer complex.

Wang et al. (2015) developed a simple and cost-effective protocol for the synthesis of quinazolines using a robust and reusable α-MnO_2_ heterogeneous catalyst. The reaction of 2-aminobenzylamines **14** with alcohols **15** in the presence of α-MnO_2_-150, TBHP (*tert*-Butyl hydrogen peroxide), chlorobenzene at 80°C produced quinazolines **16** in 59%–91% yields (Scheme 2A). (Zhang et al., 2015) The highlights of this cascade synthesis are as follows: α-MnO_2_ can be separated by centrifugation and reused with no loss in catalytic activity, and various aromatic, aliphatic, and heterocyclic alcohols are well tolerated under the reaction conditions. The authors proposed that the TBHP radicals generated on the defective sites of α-MnO_2_ probably formed TBHP-MnO_2_ adducts and carried out the transformation. Usually, catalytic activity can be increased by heteroatom doping and thermal annealing (Tilley, 2008). However, in this case, both operations failed to give the desired results. Heteroatom doping of α-MnO_2_ (with Zn, Co, Ni, Mg, and Cu) resulted in reduced yields, and annealing at temperatures >150°C induced phase reconstruction and lower activity of the catalyst due to its variable valence and the structure of the manganese oxide catalyst, which limits the use of this catalyst.

Meng et al. (2018) reported manganese oxide octahedral molecular sieve urea (OMS-2-U)-catalyzed oxidative synthesis of quinazolines under ligand-free conditions. OMS-2-U was prepared through oxidation of Mn^2+^ or reduction of Mn^4+^ using urea as the additive. This heterogeneous oxidative synthesis was carried out by reacting *N*-phenyl amidines **17** with benzyl alcohols **18** using OMS-2-U as a catalyst in toluene at 110°C under O_2_ (Scheme 2B) (Li et al., 2018a).

A variety of substituted and unsubstituted amidines **17** underwent the reaction smoothly to afford quinazolines **19**. Furthermore, benzyl alcohols **18** bearing electron-withdrawing groups gave better yields, presumably due to steric hindrance. However, electron-donating groups on benzyl alcohol **18** failed to form the desired product, which points to a shortcoming of this approach and highlights that further attention needs to be paid to the substrate scope. In addition, the enhanced surface area and crystallinity, easy separation by filtration, and reusability for at least four times makes this catalysis environmentally friendly.

OMS-2-U has excellent redox ability and serves as a catalyst in oxidation in combination with oxygen. The reaction mechanism suggested the oxidation of benzyl alcohol **18** into benzaldehyde **A** by OMS-2-U and O_2_. Subsequently, benzaldehyde **A** reacted with tautomeric amidine **B** using OMS-2-U to give intermediate **C**. Cyclization of **C** generated intermediate **D**. Finally, OMS-2-U/O_2_-catalyzed oxidative dehydrogenation of intermediate D led to the desired product **19** (Scheme 2B).

ADC reaction has emerged as a powerful tool for the sustainable synthesis of several organic molecules, since this strategy does not demand pre-functionalized substrates, hydrogen acceptors, or oxidants (Sivakumar et al., 2022). In this endeavor, Srimani *et al.* (2019) reported the first Mn-catalyzed synthesis of quinazolines **23** through the ADC. The replacement of expensive noble metal catalysts with non-toxic and earth-abundant Mn metal has made this an extremely advantageous strategy. The treatment of various 2-aminobenzyl alcohols **20** with benzonitriles **21** in the presence of Mn-pincer complex **22** bearing the *NNS*-ligand, potassium tertiary butoxide, in xylene at 140°C generated quinazolines **23** in 33%–86% yields (Scheme 2C) (Das et al., 2019).

The salient features of this strategy include: a) both electron-donating and electron-withdrawing groups in the aromatic nucleus of nitriles are well tolerated, and b) the process is atomically economical and sustainable as H_2_ and H_2_O are the only by-products of the reaction. Moreover, the evolved H_2_ can be used to convert styrene into ethylbenzene *via* Pd/C hydrogenation. However, the reaction with aliphatic nitriles such as valeronitrile yielded only 10% of the desired quinazoline **23**, which is a major limitation of this protocol, and thus the protocol requires further developments in substrate scope. Mechanistically, the reaction proceeded by the dehydrogenation of 2-aminobenzyl alcohols **20** followed by condensation *via* amidine-type intermediate **A**. Concomitant C-N bond formation and removal of water resulted in the formation of quinazolines **23** (Scheme 2C).

Similarly, Balaraman et al. (2020) proposed Mn(I)-catalyzed-direct synthesis of quinazolines **26** through an ADC strategy. The reaction of 2-aminobenzyl alcohols **20** with primary amides **24** in the presence of Mn salt and simple phosphine-free *NNN*-tridentate ligand **25** in toluene at 130°C produced quinazolines **26** in 58%–81% yields (Scheme 7). (Mondal et al., 2020) Notably, most of the catalysts used in ADC reactions possess electron-rich phosphine ligands. However, their preparation is non-trivial and requires multistep procedures and handling in an inert atmosphere. Hence, this phosphine ligand-free protocol has gained attention for sustainable syntheses. The scope of the reaction was established by varying the substitution of the amides **24**. Aliphatic, heteroaromatic, and halogen groups on aromatic ring were well tolerated. However, the electron-withdrawing groups on benzamide **24** and *trans*-cinnamide showed no reactivity under the same reaction conditions. The salient features of this protocol include: the reaction occurs even in the absence of oxidants, requires simple and abundantly available chemicals, and H_2_ and water are the only by-products of the reaction.

**SCHEME 3:**
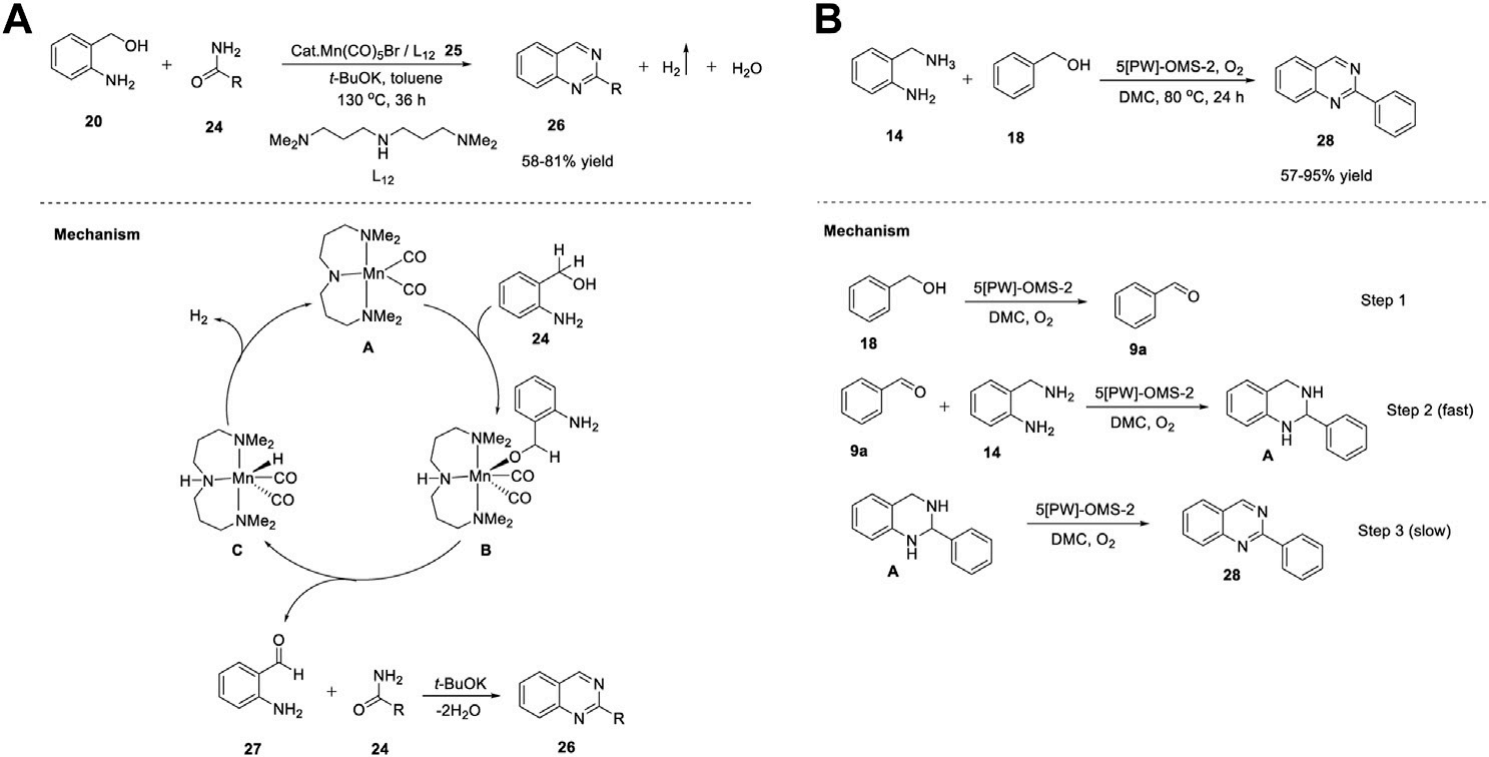
**(A)** Synthesis of quinazolines under Manganese catalysis **(B)** Synthesis of quinazolines using Manganese based nanocomposites OMS-2.

The tandem dehydrogenative annulation proceeded *via* the coordination of manganese salt Mn(CO)_5_Br and ligand L_12_ **25** in the presence of potassium *tert*-butoxide to form the active Mn catalyst **A**. Subsequently, Mn-complex **A** activated the O-H bond of 2-aminobenzyl alcohol **24** *via* metal–ligand cooperation (MLC) and generated intermediate **B**. Next, intermediate **B** underwent β-H elimination to produce 2-aminobenzaldehyde **27** and intermediate **C**. Coupling of 2-aminobenzaldehyde **27** with primary amide **24** led to the formation of quinazoline **26** *via* the elimination of water molecules. In the last step, H_2_ gas liberated from intermediate **C** *via* metal-ligand cooperation regenerated active Mn catalyst **A** (Scheme 3A).

Recently, Liu et al. (2021) developed a series of sodium phosphotungstate-doped OMS-2 catalysts (5-[PW]-OMS-2) for the synthesis of 2-phenylquinazolines. Heterogeneous catalysts previously reported for the synthesis of quinazolines still have certain disadvantages such as the use of toxic solvents and peroxides as oxidants, difficulty in catalyst preparation, and the need for unstable and expensive aldehydes as starting substrates. To overcome these difficulties, a sodium phosphotungstate-modified manganese oxide catalyst was proposed, providing a new pathway for environmentally-friendly synthesis of quinazolines **28** in a one-pot procedure. The reaction of 2-aminobenzyl amines **14** with benzyl alcohols **18** in the presence of the ([PW]-OMS-2) catalyst and O_2_ as an oxidant in dimethyl carbonate at 80°C for 24 h afforded the desired products **28** in 57%–95% yields (Scheme 3B). (Yao et al., 2021) With O_2_ as the oxidant, broad functional group tolerance and easy catalyst preparation (simple wet impregnation method) are some of the advantages of the developed strategy. However, no reaction occurred with aliphatic alcohols and two other alcohols, which leaves a mark on this protocol.

The mechanistic studies depicted in Scheme 3B reveal the formation of quinazolines **28** in three steps. In the first step, benzyl alcohol **18** was converted into benzaldehyde **9a** in the presence of (5-[PW]-OMS-2)/O_2_. Next, benzaldehyde **9a** coupled with 2-aminobenzyl amine **14** and generated intermediate **A**. Finally, the dehydrogenation of intermediate **A** resulted in 2-phenyl quinazoline **28**.

### 2.3 Iron

The second most abundant metal among all metals is iron, which is widely available in the Earth’s crust. Some superior characteristics of Fe are that it is non-toxic, cost-effective, environmentally benign, has a broad range of redox potentials, and tunes Lewis acidity based on ligands. These properties make Fe a promising catalyst candidate for organic transformations. Fe catalysts have been explored for addition (Li et al., 2018b), cross-coupling (Mako and Bayers, 2016), hydrogenation (Lane et al., 2019), substitution (Watile et al., 2019), and cycloaddition reactions (Hoyt et al., 2015).

**SCHEME 4:**
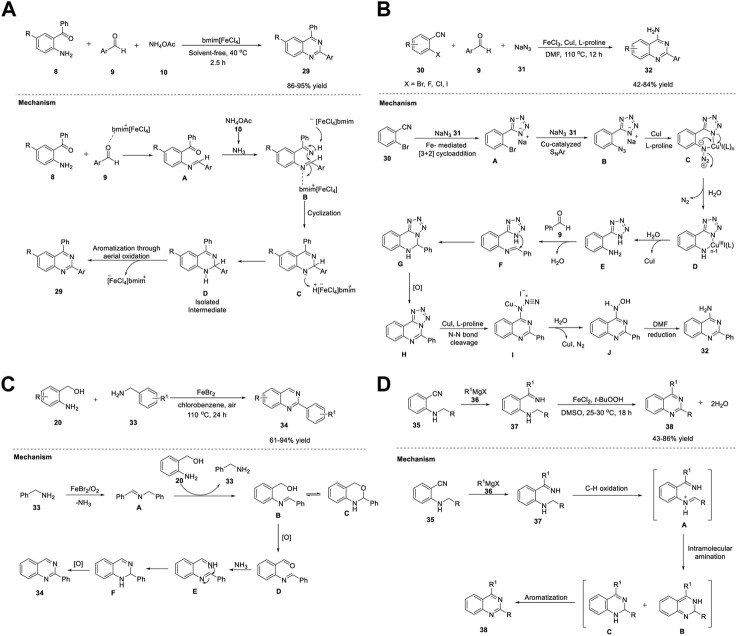
**(A)** Synthesis of quinazolines catalyzed by magnetic ionic liquid bmim [FeCl_4_] **(B)** Fe/Cu relay-catalyzed synthesis of quinazolines **(C)** FeBr_2_ catalyzed synthesis of quinazolines **(D)** Synthesis of quinazolines *via* iron-catalyzed C(sp^3^)-H oxidation.

In recent years, the multicomponent strategy has gained attention owing to its useful properties, such as that the reaction can be performed in a single step in one pot; use of ecofriendly, non-hazardous ionic liquids as solvents or catalysts; and use of solvent-free conditions sometimes (Hallett and Welton, 2011; Maeda et al., 2011; Choudhury and Singh, 2012). A new class of ionic liquids, namely, magnetic ionic liquids, has emerged as a powerful tool owing to its remarkable catalytic activity, ease of synthesis, and stability (Li et al., 2012). One such example is butylmethylimidazoliumtetrachloroferrate (bmim [FeCl_4_]) (Hayashi and Hamaguchi, 2004). The paramagnetic FeCl_4_^−^ anions in the ionic liquid respond in the presence of a magnet and hence they are named as magnetic ionic liquid. Saha et al. (2013) presented an atom-efficient, solvent-free, high yielding, multicomponent green methodology to synthesize quinazolines in a one-pot reaction of 2-aminobenzophenone **8**, benzaldehyde **9**, and ammonium acetate **10** in the presence of magnetic ionic liquid (bmim[FeCl_4_]) at 40°C for 2.5 h. Magnetic ionic liquid was efficiently used as a catalyst in multicomponent reaction (MCR) for the first time to achieve quinazolines **29** in 86%–95% yields (Scheme 4A). (Panja and Saha, 2013) Shorter reaction time and unambiguous reusability of catalyst for at least four times highlight the merit of Fe catalyst in this MCR.

Proposed tentative mechanism is shown in Scheme 4A. First, the counter cation bmim^+^ of the ionic liquid (bmim[FeCl_4_]) acts as a Lewis acid and catalyzes the condensation of the amino group of **8** with the carbonyl group of aldehyde **9** to generate intermediate **A**. Likewise, in the second step, condensation of the carbonyl group of intermediate **A** with ammonia, which is obtained from ammonium acetate **10**, results in intermediate **B**. Subsequently, FeCl_4_, the counter anionic part of the catalyst, abstracts a proton from ketimine intermediate **B** to form intermediate **C**. In the last step, the catalyst is regenerated by proton abstraction from the previous step to produce a stable intermediate **D**, which then undergoes aerial oxidation to yield the desired quinazoline **29**.

A remarkable work was put forth by Wu et al. (2015) on the synthesis of quinazolines *via* an Fe/Cu relay-catalyzed domino protocol. The reaction of *o*-halobenzonitriles **30**, benzaldehydes **9**, and sodium azide **31** (dual nitrogen source) in the presence of FeCl_3_ and CuI/L-proline in DMF at 110°C for 12 h resulted in 2-phenylquinazolin-4-amines **32** in 42%–84% yields (Scheme 4B) (Jia et al., 2015).

Commercially available starting materials and a wide substrate scope make this strategy advantageous. The reaction proceeds through the Fe-mediated [3 + 2] cycloaddition of *o*-bromobenzonitrile **30** to sodium azide **31** to generate intermediate **A**. Subsequent reaction of copper-catalyzed S_N_Ar of intermediate **A** with sodium azide **31** gave intermediate **B**, a result of the ortho substituent effect. The coordination of azide **31** with copper, followed by electrocyclization with the release of N_2_ gave Cu(III) complex **D**, which then underwent reduction with the help of a water trace in DMF, resulting in intermediate **F**. The aniline intermediate **F** underwent condensation with benzaldehyde **9** to form imine intermediate **G**. Next, intramolecular nucleophilic attack of nitrogen on imine **G** followed by oxidative dehydrogenation led to intermediate **H**. Finally, the desired product **32** was obtained *via* a copper-catalyzed denitrogenation process (Scheme 4B).

Gopalaiah et al. (2017) reported for the first time the formation of 2-aryl/heteroaryl quinazolines **34** in 61%–94% yields from 2-aminobenzyl alcohols **20** and benzylamines **33** in the presence of FeBr_2_ in chlorobenzene at 110°C under aerobic oxidation for 24 h (Scheme 4C) (Gopalaiah et al., 2017).

The key highlights of this one-pot protocol are as follows: oxygen acts as an oxidant, 2 C=N and one C-N bonds form from two different amine partners, inexpensive Fe salt acts as a catalyst, broad functional group tolerance is observed, and there is a scale-up in gram quantity without affecting yield. However, no reaction occurred with aliphatic amines, indicating a limitation in the substrate scope; otherwise, this is an outstanding work.

A plausible mechanism illustrated in Scheme 4C involves the FeBr_2_ catalyzed-oxidative self-coupling of benzylamine **33** in the presence of O_2_, leading to benzylidenebenzylamine **A** with the simultaneous removal of ammonia. Subsequently, the transamination of imine **A** with 2-aminobenzyl alcohol **20** results in 2-(*N*-benzylidene)amino benzyl alcohol **B**. Intermediate **B** exists in tautomeric equilibrium with dihydrobenzoxazine **C**, a ring form. Upon oxidation, imine intermediate **B** converts into aldehyde **D**, which then reacts with ammonia generated from benzylamine **33** in the first step to form diamine **E**. Dihydroquinazoline **F** is formed by the cyclization of **E**. Aromatization of **F** affords quinazoline **34**.

Chen *et al.* (2018) reported an efficient method for forming quinazolines **38** *via* Fe-catalyzed C (sp^3^)-H oxidation using *t*-BuOOH as the terminal oxidant (Scheme 4D) (Chen et al., 2018).

The optimized reaction conditions shown in Scheme 4D involve the treatment of easily available 2-alkylamino benzonitriles **35** with Grignard reagent **36** to form ketimine species **37**, which then leads to quinazolines **38** (43%–86% yields) in the presence of FeCl_2_ and *t*-BuOOH in DMSO at 25°C for 18 h. Adopting this strategy, heterocycles such as furan, thiophene, and pyridine can be readily installed at the 2- position of quinazoline **38**. The synthetic strategy involves the addition of Grignard or Organolithium reagent **36** to *o*-alkylamino benzonitriles **35** to form *o*-alkylamino N-H ketimines **37**. Subsequently, C(sp^3^)-H oxidation of the α-proton of the aminoalkyl group generates imine or iminium species **A**. Ring closure *via* nucleophilic attack of iminium **A** then results in dihydroquinolines **B** and **C**. Aromatization through oxidation affords the desired quinazoline **38** (Scheme 4D).

**SCHEME 5:**
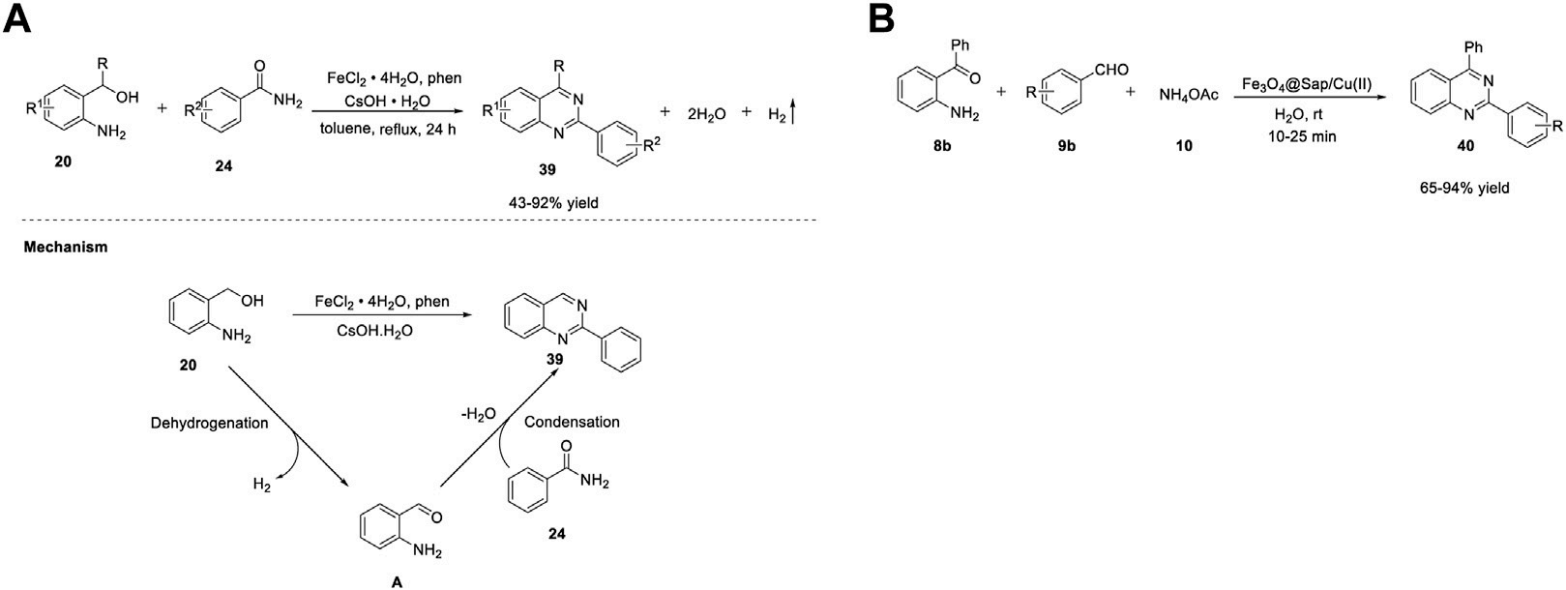
**(A)** Synthesis of quinazolines by Fe-catalyzed acceptorless dehydrogenative coupling **(B)** Synthesis of quinazolines using Fe_3_O_4_@Sap/Cu(II) nanocatalyst.

Li *et al.* (2021) demonstrated the efficient synthesis of quinazolines **39** using the ADC strategy. Reaction of (2-aminophenyl)methanols **20** with benzamides **24** in the presence of FeCl_2_·4H_2_O, phenanthroline (ligand) in CsOH·H_2_O, and toluene at 130°C for 24 h delivered quinazolines **39** in 43%–92% yields (Scheme 13). (Zhang et al., 2021) A broad substrate scope and hydrogen and water as the only byproducts of the reaction are the merits of this approach. The proposed mechanism involves the one-pot dehydrogenation of (2-aminophenyl)methanol **20** into aldehyde **A,** followed by condensation with benzamide **24** in the presence of a base to produce quinazoline **39** (Scheme 5A).

Heterogeneous catalysis has been used as a complementary protocol to homogenous catalysis. Kazemnejadi et al. (2021) developed a novel magnetically recoverable green nanocatalyst Fe_3_O_4_@Sap/Cu(II) for accessing quinazolines **40**. Saponin belongs to a group of plant amphipathic glycosides with steroid/triterpenoid aglycones and is soluble in both water and fats; hence, it is a good choice in a phase transfer protocol (Güçlü-Üstündağ and Mazza, 2007). Immobilization of saponin as a green shell on Fe_3_O_4_ NPs (nanoparticles) as a magnetic solid support is a promising strategy. Furthermore, water is also a green solvent that has many advantages in organic synthesis such as easy product separation, high reaction rates, and cheap solvent. The one-pot cyclocondensation of aromatic aldehydes **9b** with 2-aminobenzophenone **8b** and ammonium acetate **10** catalyzed by Fe_3_O_4_@Sap/Cu(II) in water at room temperature for 10–25 min afforded quinazolines **40** in 65%–94% yields (Scheme 5B). (Kazemnejadi et al., 2021) High selectivity of substrates, shorter reaction times, and reusability of the catalyst for at least six times are the highlights of this protocol.

### 2.4 Cobalt

Cobalt is an inexpensive and less toxic metal that exhibits high chemoselectivity and variable oxidation states. Among the first-row transition metals, it is one of the most attractive candidates for catalysis. Hence, it is used to carry out various transformations, such as coupling reactions (Bottaro and Madsen, 2019), hydrofunctionalization (Yang et al., 2019a), hydrogenation (Viereck et al., 2020), and cycloaddition (Ding and Yoshikai, 2019).

**SCHEME 6:**
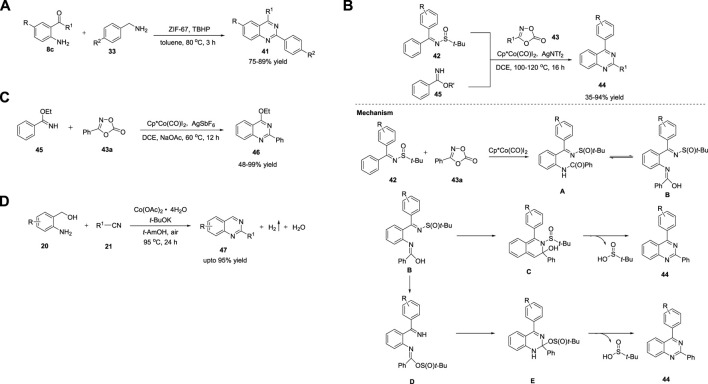
**(A)** Cobalt imidazolate framework ZIF-67 catalyzed synthesis of quinazolines **(B)** Cp*Co(CO)I_2_ catalyzed synthesis of quinazolines **(C)** Synthesis of quinazolines *via* Co catalyzed [4 + 2] cycloaddition **(D)** Co-catalyzed ADC coupling for the synthesis of quinazolines.

Metal-organic frameworks (MOFs) are defined as extended porous materials composed of metal ions or metallic clusters and multifunctional organic linkers (Zhang et al., 2009). They are applied in chemical sensors, biomedical imaging, catalysis, etc. (Getman et al., 2012) Zeolite imidazolate frameworks (ZIFs) have emerged as a new subclass of MOFs. Phan et al. (2015) reported efficient heterogeneous catalysis for the synthesis of quinazolines **41** using a cobalt zeolite imidazolate framework (ZIF-67). The reaction of 2-aminobenzophenone **8c** with benzyl amine **33** in the presence of the ZIF-67 catalyst in TBHP at 80°C for 180 min delivered the desired quinazolines **41** in 75%–89% yields (Scheme 6A). (Truong et al., 2015) Low temperature, catalyst reusability, and good functional group tolerance are the key features of this methodology.

The C-H functionalization of arenes has been employed as a promising protocol for the synthesis of functional organic molecules and natural products because of the ubiquity of C-H bonds. Direct C-H activation of arenes usually depends on second- and third-row transition metals (McMurray et al., 2011). Li et al. (2016) reported the first synthesis of quinazolines *via* the C-H activation pathway using a Co catalyst. The optimized conditions involve the reaction of *N*-sulfinylimines **42** and benzimidates **45** bearing a directing group with dioxazolone **43**, a synthon of nitriles, in the presence of Co catalyst Cp*Co(CO)I_2_ and AgNTf_2_ (an activator) in DCE at 100°C–120°C for 16 h produced quinazolines **44** in 39%–94% yields (Scheme 6B). (Wang et al., 2016a) Various substituents introduced on the substrates were well tolerated with high regio- and mono/diselectivity. Starting with different arenes, two types of quinazolines **44** were synthesized. In the first approach, C-H activation is aided by an autocleavable N-S bond. In the second approach, the stability of benzimidates **45** is the driving force for the formation of quinazolines **44**. The halogen group in quinazoline derivatives **44** should allow further transformations. However, alkyl-substituted dioxazolone **43** reacted with a lower yield.

The mechanism depicted in Scheme 16 for the coupling reaction of *N*-sulfinylimine **42** and dioxazolone **43a** revealed that the initial Co(III)-catalyzed C−H activation followed by amidation generates amide intermediate **A**, which undergoes 6π-electrocyclization *via* imidic acid tautomer **B** to deliver dearomatized intermediate **C**. Quinazoline **44** is formed *via* the elimination of *tert*-butanesulfinic acid. Simultaneously, **B** undergoes nucleophilic attack at the sulfinyl group to produce an ester intermediate **D**, which upon intramolecular nucleophilic addition forms **E**, and subsequent elimination of *tert*-butanesulfinic acid affords product **44**. In both cases, the post-amidation process was uncatalyzed.

In the same year, Glorius *et al.* (2016) documented Co-catalyzed formal [4 + 2] cycloaddition of arenes with rarely explored free imines and dioxazolones for facile access to quinazolines **46**. The reaction of ethyl benzimidate **45** with dioxazolone **43** in the presence of Cp*Co(CO)I_2_ and AgSbF_6_ in NaOAc and DCE at 60°C for 12 h produced quinazolines **46** in 48%–99% yields (Scheme 6C) (Wang et al., 2016b). This tandem C-H amidation and cyclization is uniquely catalyzed by the Co complex and represents a complementary process to multi-substituted quinazoline synthesis. The excellent substrate scope and reactivity are attributed to the strong Lewis acidity and high sensitivity to the steric hindrance of the Co(III) catalyst. Easily available substrates and high yields are other advantages of this method.

Recently, Lin et al. (2022) reported an efficient one-pot strategy for Co-catalyzed formation of quinazolines **47** *via* ADC. The dehydrogenative cyclization of 2-aminoaryl alcohols **20** and nitriles **21** in the presence of Co(OAc)_2_·4H_2_O and *t*-BuOK in *tert*-AmOH in air at 95°C for 24 h produced quinazolines **47** in up to 95% yields (Scheme 6D). (Hao et al., 2022) The salient features of this protocol are as follows: mild reaction conditions, simple operation, ligand-free, wide substrate compatibility, and cost-effective catalyst.

### 2.5 Nickel

**SCHEME 7:**
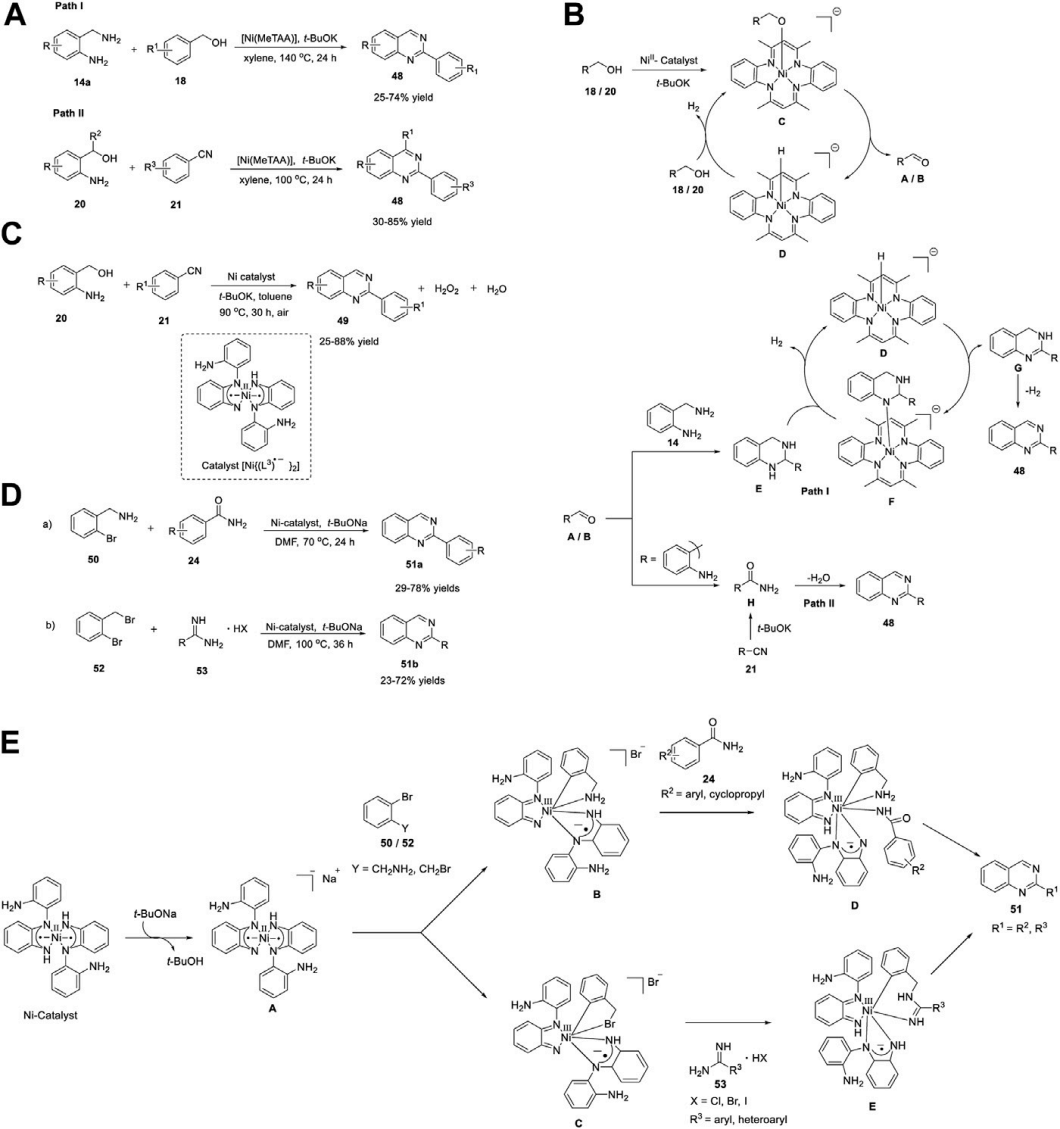
**(A)** Synthesis of quinazolines *via* Ni-catalyzed acceptorless dehydrogenative coupling **(B)** Plausible mechanism for the construction of quinazolines through ADC reaction **(C)** Synthesis of quinazolines *via* Ni catalyzed dehydrogenative coupling **(D)** Synthesis of quinazolines *via* Ni-catalyzed C-N cross coupling **(E)** Proposed mechanism for Ni-catalyzed synthesis of quinazolines.

Ni belongs to the precious metals group, accompanied by Pt and Pd. Ni complexes are highly reactive organometallic species that exhibit multiple oxidation states, are less expensive than other precious metals, and have a strong affinity for unsaturated systems. Numerous Ni-catalyzed reactions have been reported, such as cyclization (Fujimoto et al., 2019), oligomerization (Li et al., 2017), cross-coupling reactions (Muto et al., 2015), and cascade reactions (Chen et al., 2019). Paul *et al.* (2018) reported an environmentally benign protocol for the production of quinazolines **48** with 25%–85% yields through ADC of a) 2-aminobenzylamine **14a** with benzyl alcohol **18** and b) 2-aminobenzylalcohol **20** with benzonitrile **21** in the presence of a Ni catalyst [Ni(MeTAA)] with a macrocyclic ligand (tetramethyltetraaza[14]annulene (MeTAA)) in *t*-BuOK and xylene at 100°C–140°C for 24 h (Scheme 7A) (Parua et al., 2018).

The salient features of this strategy include easily available starting precursors, use of an inexpensive, Earth-abundant, easily prepared Ni catalyst, high atom efficiency, non-toxic byproducts such as H_2_ and water, and broad functional group tolerance in both pathways, including hetero substituents. Notably, addition of styrene as a sacrificial hydrogen acceptor *via* path **I** increased the product yield, whereas path **II** did not require any sacrificial hydrogen acceptor.

A stepwise reaction mechanism has been proposed by the authors (Scheme 7B). The first step is the dehydrogenation of alcohols **18** and **20** to the corresponding aldehydes **A** and **B**, in both pathways. This proceeds *via* the following sub-steps: initial deprotonation of alcohols (benzyl alcohol **18** in path I and 2-aminobenzylalcohol **20** in path **II**), formation of Ni-alkoxy intermediate **C,** and finally dehydrogenation. In the second step, benzaldehyde **A** (Path **I**) undergoes cyclocondensation with 2-aminobenzylamine **14** to generate intermediate **E**. In the last step, [Ni(MeTAA)]-catalyzed dehydrogenation of **E** affords the desired product **48**. Likewise, the base-promoted cyclocondensation of 2-aminobenzaldehyde **B** with benzamide (generated *in situ* from benzonitrile **21**) affords quinazoline **48**.

In this endeavor, Paul *et al.* (2019) developed a new bio-mimetic method for the production of poly-substituted quinazolines **49** through dehydrogenative condensation. Coupling of 2-aminobenzylalcohols **20** with nitriles **21** catalyzed by diradical nickel (II)-complex [Ni^II^{(L^3^) ^•−^}_2_] having two diamine ligands in the presence of *t*-BuOK and toluene at 90°C for 30 h gave the desired quinazolines **49** in 25%–88% yields (Scheme 7C). (Chakraborty et al., 2019) In the Ni catalyst, two one-electron oxidized-tridentate *N*-(2-aminophenyl)benzene-1,2-diamine ligands (L^3^) were coordinated with the Ni(II) center in a bidentate manner, where all or a part of the redox process takes place at the ligands.

In the same year, Paul et al. (2019) demonstrated the C-N cross coupling reaction of a) 2-bromobenzylamine **50** with benzamides **24** and b) 2-bromobenzylbromide **52** with amidines **53** to form the corresponding multi-substituted quinazolines **51** in 23%–78% yields, catalyzed by singlet diradical Ni(II)-catalyzed [Ni^II^{(L^3^) ^•−^}_2_] in the presence of NaO^*t*^Bu and DMF at 70°C–100°C for 24–36 h, respectively (Scheme 7D). (Sikari et al., 2019) Under the optimized conditions, different electron-donating and electron-withdrawing groups on benzamide **24** produced significant yields of desired product **51a**. The low yields (29%–38% yield) obtained with heteroarylamides **24** might be due to their ability to coordinate with the catalyst, thereby inhibiting the catalytic cycle. Furthermore, electron-donating- and electron-withdrawing-substituted benzamidines **53** were suitable candidates for C-N cross-coupling, providing better yields. However, cyclopropyl amidine **53** afforded quinazoline **51b** in a lower yield (23% yield), indicating a limitation in the substrate scope of both approaches. Easy preparation, cost-effectiveness, and air stability are promising features of the Ni catalyst used in this straightforward strategy, which offers an elegant alternative to expensive transition metal catalysts. Furthermore, during catalytic turnover, the cooperative involvement of Ni and ligand-centered redox processes provides an alternative approach for energetically demanding Ni-centered redox processes.

Insight into cross-coupling was obtained by performing mechanistic studies, as shown in Scheme 7E. The reaction mechanism begins with the formation of active catalyst **A** *via* deprotonation of the complex. Anionic -NH deprotonated species **A** facilitates the oxidative addition of aryl halides *viz.* 2-bromobenzylamine **50** and 2-bromobenzylbromide **52** in a synergistic fashion *via* Ni(II)/Ni(III) and {(L3)•2−}/{(L3)−} redox couples to form intermediates **B** and **C**, respectively. Consequently, *N*-arylation followed by intramolecular cyclization of intermediates **D** and **E** affords quinazolines **51**.

### 2.6 Copper

Copper is an inexpensive, non-toxic, and Earth-abundant metal that catalyzes reactions involving one- and two-electron mechanisms. Cu can activate terminal alkynes. Cu complexes/salts undergo several transformations such as cycloaddition (Brittain et al., 2016), oxidation (Kathiravan et al., 2019), radical (Gu et al., 2020), and coupling reactions (Li et al., 2020).

**SCHEME 8:**
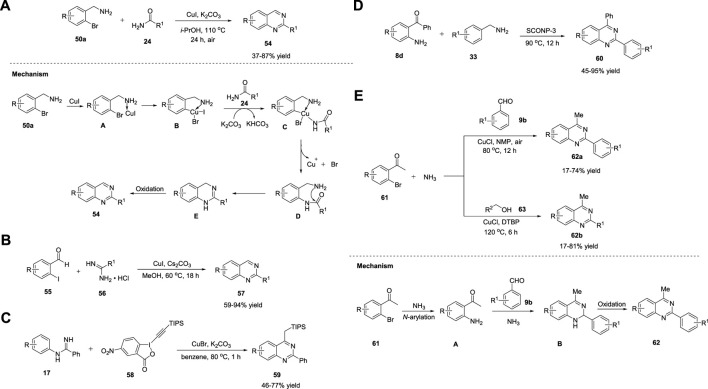
**(A)** Proposed mechanism for Ni-catalyzed synthesis of quinazolines **(B)** Proposed mechanism for Ni-catalyzed synthesis of quinazolines **(C)** CuBr catalyzed synthesis of quinazolines *via* C-H alkynylation **(D)** CuO NPs catalyzed synthesis of quinazolines **(E)** CuCl catalyzed synthesis of quinazolines.

Fu et al. (2010) reported Ullmann-type coupling for the Cu-catalyzed synthesis of quinazolines **54** in 37%–87% yields from readily available 2-bromophenyl methylamines **50a** and amides **24** in the presence of Cu(I) and K_2_CO_3_ in *i*-PrOH in air at 110°C for 24 h (Scheme 8A). (Wang et al., 2010) The *ortho*-substituent effect of the amino group in **50** was the driving force for the absence of the ligand in this cascade reaction. All substrates were well tolerated. However, aliphatic amides failed to yield any product. Easily available substrates, ligand- or additive-free reaction, and air as green oxidants are some of the key advantages of this protocol. Sequential Ullmann-type coupling begins with the coordination of (2-bromophenyl)methylamine **50a** with Cu(I) ions, resulting in intermediate **A**, which upon oxidative addition gives intermediate **B**. Intermediate **C** is formed by the reaction of **B** with amide **24** in the presence of K_2_CO_3_. Subsequently, reductive elimination of **C** leads to *N*-arylated species **D**, leaving the copper catalyst. Intramolecular dehydrative cyclization of **D** followed by air-promoted aromatization provides quinazoline **54** (Scheme 8A).

Likewise, Truong et al. (2010) proposed a ligand-free Cu-catalyzed Ullmann condensation for highly functionalized quinazolines **57**. The reaction of *o*-iodobenzaldehydes **55** and amidine hydrochlorides **56** in the presence of Cs_2_CO_3_ in methanol at 60°C for 18 h afforded the desired product **57** in 59%–94% yields (Scheme 8B), (Truong and Morrow, 2010) while using *o*-bromobenzaldehyde gave only 5% yield, which is a limitation to the substrate scope. Mild reaction conditions, one-pot methodology, and ligand-free conditions are the striking features of this protocol.

In the same year, Ohno *et al.* (2010) demonstrated the Cu-catalyzed synthesis of quinazolines **58** from ortho-unfunctionalized aniline derivatives **17** *via* C-H alkynylation and cyclization. The reaction of *N*-phenylbenzamidines **17** with 5-nitro-1-[(triisopropylsilyl)ethynyl]-1,2-benziodoxol-3(1H)-one **58** (alkyne source) in the presence of a catalytic amount of CuBr in K_2_CO_3_ and benzene at 80°C for 1 h gave quinazolines **59** in 46%–77% yields (Scheme 8C). (Ohta et al., 2010) Furthermore, TIPS could be cleaved by the treatment of **59** with TBAF in THF-AcOH (20:1) at room temperature to yield quinazolines. Direct synthesis and commercially available starting materials are the notable features of this protocol. The formation of highly bipolar byproducts is the reason for the moderate product yields.

Metal NPs have gained attention as semi-heterogeneous catalysts (Shiju and Guliants, 2009). Copper oxide NPs (CuO NPs) belong to this class. Wang *et al.* (2010) disclosed a novel protocol for the production of a series of quinazolines **60** in 45%–95% yields *via* the reaction of 2-aminobenzophenones **8d** and benzylic amines **33** catalyzed by CuO NPs supported on kaolin (SCONP-3) at 90°C for 12 h (Scheme 8D). (Zhang et al., 2010) Both electron withdrawing and electron donating groups favored the reaction with excellent yields. Inexpensive, stable, and reusable for at least four times are the benefits of this heterogeneous catalyst.

Hua et al. (2012) developed two efficient approaches to produce multi-substituted quinazolines **62a/b**: a three-component one-pot reaction of I) o-bromo aromatic ketones/aldehydes **61**, aromatic aldehydes **9b**, and ammonia water, and II) o-bromo aromatic ketones/aldehydes **61**, primary alcohols **63**, and ammonia water in the presence of CuCl, NMP, and air/DTBP, respectively, at 80°C–120°C for 6–12 h (Scheme 8E). (Ju et al., 2012) Under optimized conditions, quinazolines **62a/b** were obtained in 17%–81% yields using ammonia water as the nitrogen source and air/DTBP as the oxidant. A variety of substituents, including electron-donating and electron-withdrawing groups, were well tolerated. The advantages of this approach include easy availability of precursors and use of air as oxidant. Mechanistically, the Ullmann-type amination reaction of *o*-haloacetophenones **61** with ammonia generated intermediate **A**, which upon cyclocondensation with aldehyde **9b** and ammonia followed by aerobic oxidation gave desired product **62a/b** (Scheme 8E).

**SCHEME 9:**
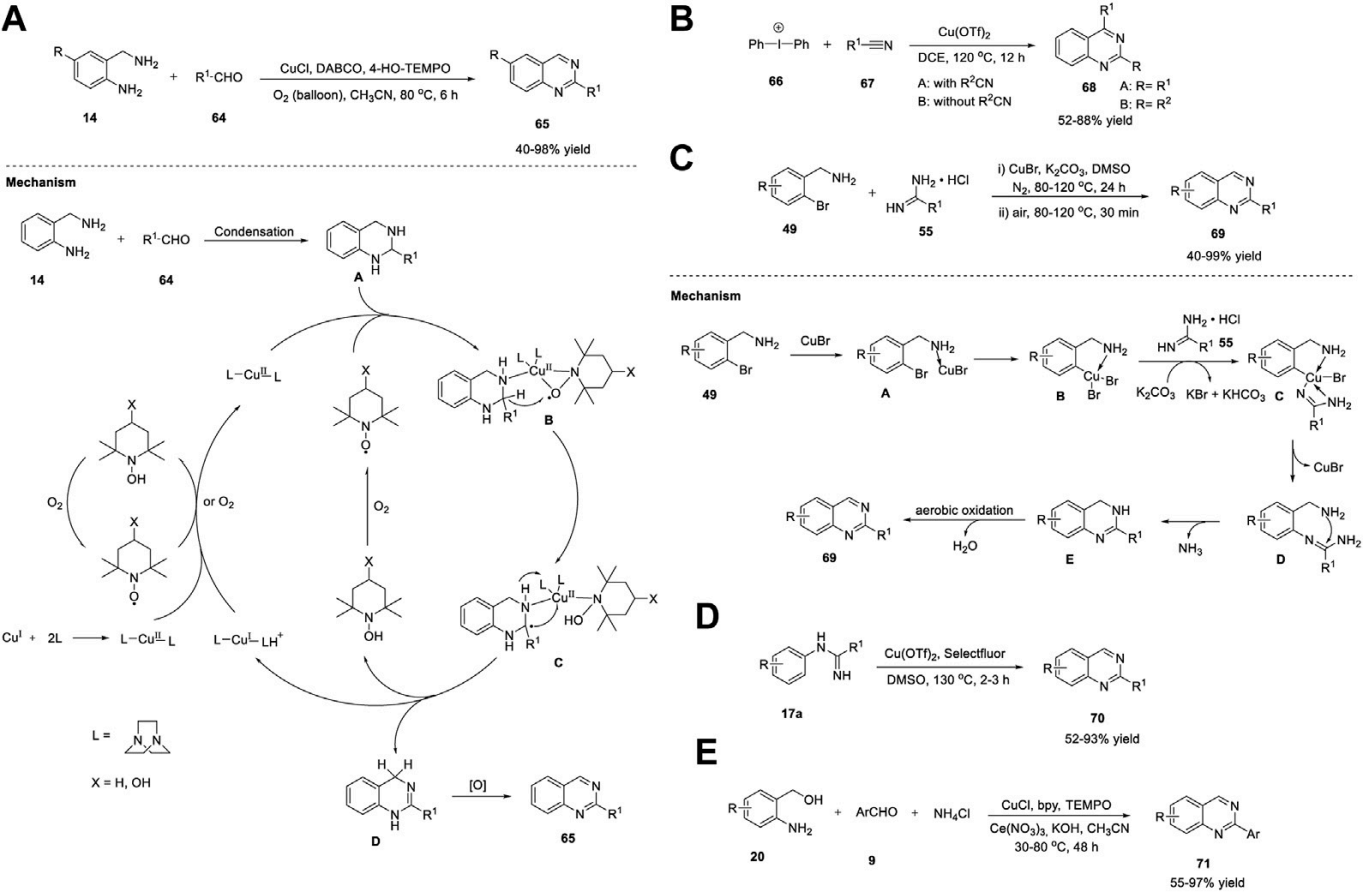
**(A)** CuCl/DABCO/TEMPO- catalyzed synthesis of quinazolines **(B)** Cu(OTf)_2_ catalyzed synthesis of quinazolines *via* [2 + 2+2] annulation **(C)** CuBr catalyzed synthesis of quinazolines **(D)** Cu-catalyzed annulation of amidines for the synthesis of quinazolines **(E)** CuCl catalyzed cascade synthesis of quinazolines.

Yu et al. (2012) explored aerobic oxidative synthesis using the Cu/*N*-ligand/TEMPO catalytic system, the first example of its kind, in the synthesis of heterocycles and quinazolines in particular. The reaction of 2-aminobenzylamines **14** with aldehyde **64** in the presence of CuCl/DABCO/4-HO-TEMPO in acetonitrile and oxygen as the terminal oxidant at 80°C for 6 h afforded quinazolines **65** in 40%–98% yields (Scheme 9A). (Han et al., 2012) The aerobic oxidative reaction could be scaled up to one g without any difficulty. Heterocyclic aldehydes **64** such as 3-picolylaldehyde and 2-furylaldehyde also afforded corresponding quinazolines **65** in good yields. However, alkyl aldehydes **64** gave lower yields of the product, which was the only limitation in the substrate scope; otherwise, this is an outstanding work.

Oxidative dehydrogenation proceeded *via* the formation of the Cu^II^-(DABCO)_2_ complex through the oxidation of Cu^I^-(DABCO)_2_ by oxygen or TEMPO. Further coordination of the Cu^II^-(DABCO)_2_ complex with the *N*-atom of tetrahydroquinazoline **A** and TEMPO resulted in η-2 manner intermediate **B** (Caneschi et al., 1988). The transfer of the benzylic hydrogen atom in **B** to TEMPO *via* a hydrogen abstraction step resulted in radical-TEMPOH Cu species **C**. The benzyl radical then underwent oxidation *via* Cu(II)-mediated inner-sphere electron transfer, resulting in the corresponding carbocation, which then underwent deprotonation to give dihydroquinazoline **D**, with the simultaneous formation of Cu(I) species and TEMPOH. Subsequently, TEMPOH was autoxidized into TEMPO, which then reoxidized the Cu(I) species into Cu^II^-(DABCO)_2_. In the last step, intermediate **D** was oxidized again to form desired product **65** (Scheme 9A).

Chen *et al.* (2013) presented an efficient approach for the one-pot synthesis of multisubstituted quinazolines **68** *via* [2 + 2 + 2] cascade annulation with diaryliodonium salts **66** and nitriles **67** in the presence of Cu(OTf)_2_ in DCE at 120°C for 12 h (Scheme 9B). (Su et al., 2013) Various aliphatic and aromatic nitriles **67** worked well and produced quinazolines **68** in 52%–88% yields. Electrophilic annulation involves the use of readily available starting materials and encompasses a wide range of functional groups. The reaction proceeded with the addition of two different nitriles in sequence to give regioselective products. However, ethyl cyanoformate and diethyl cyanophosphate did not undergo annulation owing to electron deficiency.

Cheng et al. (2013) reported a novel Cu-catalyzed cascade method for the production of quinazolines. The optimized reaction conditions included (2-bromophenyl)methylamines **49** and amidine hydrochlorides **55** as substrates, CuBr as the catalyst, K_2_CO_3_ as the base, and DMSO as the solvent at 100°C in N_2_ for 24 h and then in air for 30 min to furnish quinazolines **69** in 40%–99% yields (Scheme 9C). (Liu et al., 2013) The salient features of this protocol are as follows: inexpensive copper catalyst, air as a green oxidant, and broad functional group tolerance. The proposed mechanism begins with the coordination of (2-bromophenyl)methylamine **49** with CuBr to generate complex **A**, which upon oxidative addition provides **B**. Base-mediated complexation of **B** with amidine gives **C**. Reductive elimination of **C** affords *N*-arylation species **D**, leaving the catalyst. Subsequently, intramolecular nucleophilic attack of the amino groups on the carbon of the amidine group generates **E**, resulting in the loss of NH_3_. Finally, the aerobic oxidation of **E** results in the desired quinazoline **69** (Scheme 9C).

In this study, Zhang et al. (2013) illustrated a facile Cu-catalyzed synthesis of quinazolines **70** from *ortho*-unfunctionalized aniline *viz.* amidines **17a** and DMSO, DMA, DMF, TEMDA, or NMP *via* direct oxidative amination of N-H bonds and C (sp^3^)-H bonds, followed by intramolecular C-C bond formation reactions. Quinazolines were obtained in 52%–93% yields by employing amidine as a nitrogen source, sp^3^ carbon in DMSO as a one-carbon synthon, Cu(OTf)_2_ as the catalyst, and Selectfluor as an oxidant at 130°C for 2–3 h (Scheme 9D). (Lv et al., 2013) This is the first example of a C-N bond formation reaction between the N-H bond of amidine and the methyl C (sp^3^)-H bonds of solvents. The broad substrate scope and high selectivity of the annulation reaction towards different C(sp^3^)-H bonds and easy preparation of amidines make this an attractive method. However, cyclic amidines failed to yield the desired product, which is a limitation of this approach.

Wu et al. (2013) reported the first example of the Cu-catalyzed reaction of (2-aminophenyl)methanols **20** with aldehydes **9** in the presence of CuCl, bipyridine, TEMPO, and cerium nitrate hexahydrate–ammonium chloride in KOH and acetonitrile at 30°C–80°C for 48 h, leading to the formation of quinazolines **71** in 55%–97% yields (Scheme 9E). (Chen et al., 2013) The reaction proceeded smoothly and tolerated a wide variety of functional groups. Furthermore, the reaction could be easily scaled up to gram quantities, which is a noteworthy advantage.

**SCHEME 10:**
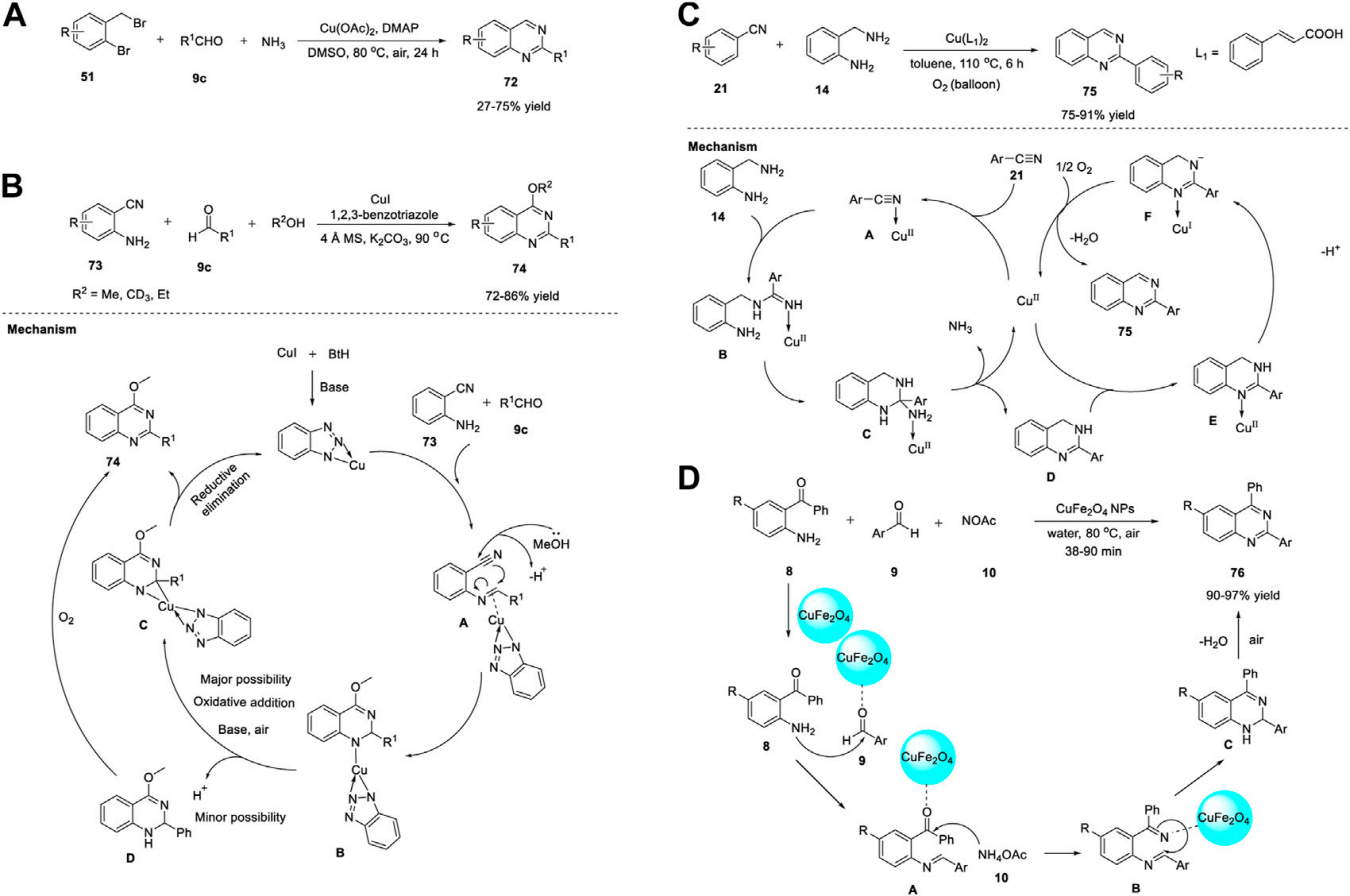
**(A)** Cu(OAc)_2_ catalyzed synthesis of quinazolines **(B)** Cu-benzotriazole catalyzed synthesis of quinazolines **(C)** Cu-catalyzed cascade coupling and aerobic oxidation for the synthesis of quinazolines **(D)** CuFe_2_O_4_ NPs catalyzed synthesis of quinazolines.

Similarly, Fan et al. (2013) developed a simple and economical synthesis method of quinazolines **72** through the Cu-catalyzed cascade reaction of 2-bromobenzylbromides **51**, aldehydes **9c**, and aqueous ammonia in the presence of Cu(OAc)_2_, DMAP, and DMSO at 80°C in air for 24 h (Scheme 10A). (Fan et al., 2014) Sequential cupric acetate-catalyzed amination, condensation, intramolecular nucleophilic cyclization, and aromatization led to the roduction of quinazoline **72** in 27%–75% yields. The structural diversity of the products and easy availability of the starting materials are the benefits of this approach.

In a study, Ahmed et al. (2014) reported an efficient approach to produce *o*-protected-4-hydroxyquinazolines **74** *via* copper-benzotriazole (Cu-BtH)-catalyzed intramolecular electrophilic cyclization. The reaction proceeded *via* the treatment of 2-aminobenzonitriles **73** with aldehydes **9** in the presence of Cu(I)-benzotriazole, K_2_CO_3_, and 4 Å molecular sieves in alcohol at 90°C, providing quinazolines **74** in 72%–86% yields (Scheme 10B). (Battula et al., 2014) Use of Cu in catalytic amounts is the benefit of this protocol despite long reaction time and relatively high temperature. In addition, this is the first approach for synthesis of *o*-protected-4-hydroxyquinazolines **75** through a monocyclic system *via* a bicyclic intermediate. All types of aldehydes **9c**, such as aryl, alkyl, and heteroaryl aldehydes, were converted into the corresponding quinazolines **74** in excellent yields.

A plausible reaction mechanism proposed by the authors is shown in Scheme 10B. Initially, 2-aminobenzonitrile **73** forms an *N*-arylimine with aldehyde **9c**. Complex **A** is formed by the coordination of the imine with the BtH-ligated Cu. Simultaneous intramolecular electrophilic cyclization and oxidative addition of alcohols gives complex **B**, which is converted to complex **C** through oxidative addition in the presence of a base and air. In the last step, the reductive elimination of **C** leads to the formation of quinazoline **74**. An additional pathway is presumed to explain the presence of compound **D**, wherein the complex in the H^+^ environment forms dihydroquinazoline, which then undergoes oxidation to afford quinazoline **74**.

Li et al. (2014) reported a green method for the synthesis of quinazolines **75** through Cu-catalyzed cascade coupling and aerobic oxidation without the need for additives or bases. The optimized reaction conditions involve the coupling of benzonitrile **21** with 2-aminobenzylamine **14** in the presence of Cu^II^(L_1_)_2_ and cinnamic acid as the ligand, under air in toluene at 110°C for 6 h, affording quinazolines **75** in 75%–91% yields (Scheme 10C). (Li et al., 2014) The key features of this strategy include: a) Cu acts as a dual catalyst for both cascade coupling and aerobic oxidation, b) O_2_ acts as a terminal oxidant, c) base- or additive-free reaction, d) good substrate specificity, and e) mild reaction conditions in a one-pot fashion.

A tentative mechanism as depicted in Scheme 10C begins with the activation of nitrile **21** by Cu(II) to provide an intermediate **A**. Nucleophilic addition of **A** with 2-aminobenzylamine **14** gives **B**. Intermediate **C** is formed by the intramolecular cycloaddition of **B** followed by removal of Cu^II^(L_1_)_2_ and NH_3_ furnished the compound **D**. Then, the nitrogen atom of tertiary amine **D** coordinates to Cu^II^(L_1_)_2_ to form **E** *via* a ligand-exchange reaction. Subsequently, intermediate **F** is formed by the deprotonation of **E**, with simultaneous liberation of the desired product **75** and reoxidation of the Cu(I) complex to the Cu(II) complex.

Magnetic NPs have gained considerable attention owing to their unique features such as a) ease of preparation from non-toxic substances, b) simple separation by means of an external magnet, c) cost-effective and controllable fabrication, d) low catalyst leaching compared to that of other material-supported catalysts, and e) use of eco-friendly solvents. Baghbanian et al. (2014) reported a one-pot method for the synthesis of quinazoline derivatives **75** in the presence of CuFe_2_O_4_ NPs as reusable catalysts in water (Scheme 10D). The tandem cyclization reaction between 2-aminoketone **8** with aldehyde **9** and ammonium acetate **10** in the presence of CuFe_2_O_4_ NPs in water at 80°C for 38–90 min produced quinazolines **76** in 90%–97% yields (Scheme 10D). (Baghbanian and Farhang, 2014) The major benefits of this technique are the simplicity of product/substrate extraction from the catalyst, reusability of the catalyst for at least five times, and chemoselectivity. The reaction mechanism is initiated by the coordination of the clean catalyst, CuFe_2_O_4_ NPs, with the carbonyl groups of 2-aminoketone **8** and aldehyde **9**. This coordination results in increased electrophilicity of the carbonyl carbons and thus promotes the nucleophilic attack of the amine group in **8** and ammonium acetate **10**. Subsequently, the condensation of aldehyde **9** with amine **8** generates aldimine **A**. The attack of ammonium acetate **10** on the keto group of benzophenone leads to the formation of ketimine **B**. Subsequent ring closure followed by aromatization through dehydration in conjunction with O_2_ from the air gives desired product **76** (Scheme 10D).

**SCHEME 11:**
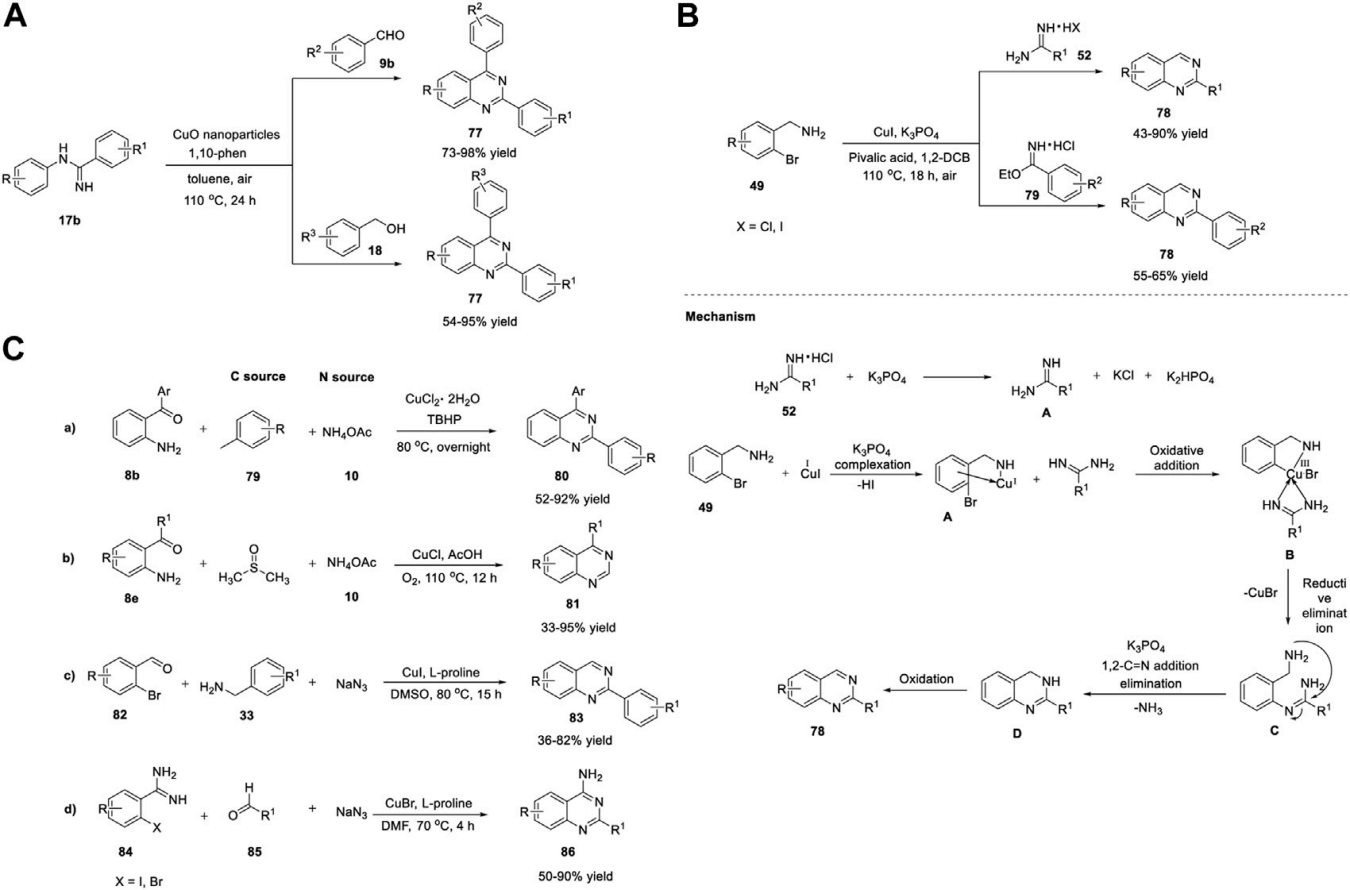
**(A)** Synthesis of quinazolines *via* CuO NPs catalyzed oxidative coupling **(B)** Cu(I)-catalyzed *N*-arylation for the synthesis of quinazolines **(C)** Cu-catalyzed amination/one-pot method for the synthesis of quinazolines.

Likewise, Zhang et al. (2014) identified CuO NPs as inexpensive catalysts for producing quinazolines **77** *via* aerobic oxidative coupling (Scheme 11A). The treatment of *N*-arylamidines **17b** with benzaldehyde **9b** or benzyl alcohol **18** in the presence of CuO NPs and 1,10-phenanthroline in toluene in air at 110°C for 24 h led to the formation of quinazolines **77** in 54%–98% yields (Scheme 11A). (Zhang et al., 2014b) CuO NPs have proven to be more effective heterogeneous catalysts than the other CuO nanocatalysts and commercial CuO, exhibiting remarkable efficiency. The straightforward synthetic protocol, inexpensiveness, recyclability of the Cu catalyst, high generality, and good functional group tolerance are the benefits of this oxidative coupling approach.

Beifuss *et al.* (2014) demonstrated two efficient methods for the synthesis of 2-substituted quinazolines **78** *via* Cu-catalyzed domino intermolecular *N*-arylation/intramolecular nucleophilic substitution (Scheme 11B).

The first method involved one-pot reaction between 1-(2-bromophenyl)methanamines **49** and amidines **52**/imidates **79** using Cu(I) as catalyst, K_3_PO_4_ as base, and pivalic acid as an additive in 1,2-dichlorobenzene at 110°C for 18 h under aerial O_2_ as an oxidant to produce quinazolines **78** in 43%–90% yields (Scheme 11B). (Omar et al., 2014) External oxidant-free, ligand-free, broad substrate scope of both 1-(2-bromophenyl)methanamines **49** and amidines **52**, and inexpensive Cu catalyst are the promising features of this protocol. Mechanistically, coordination of Cu(I) with 1-(2-bromophenyl)methanamine **49** in the presence of a base gives intermediate **A**. Oxidative addition of **A**, followed by complex formation with the amidine, generates **B**, which then undergoes reductive elimination to produce **C** and CuBr. Furthermore, intramolecular 1,2-addition of an amine group to the CN group of the amidine species in the presence of a base delivers the cyclized species **D**, with loss of ammonia. Finally, Cu-catalyzed oxidation of **D** with O_2_ in air results in quinazoline **78** (Scheme 11B).

Wang et al. (2015) reported a novel Cu-catalyzed double oxidative C-H amination of methylarenes **79** for the formation of 2-arylquinazolines **80**. The optimized reaction conditions involved the treatment of 2-aminobenzoketones **8b** with ammonium acetate **10** (N source) and toluene **79** as the solvent and a reagent in the presence of CuCl_2_·2H_2_O as the catalyst and TBHP as an oxidant at 80°C to generate quinazolines **80** in 52%–92% yields (Scheme 11Ca) (Liu et al., 2015).

**SCHEME 12:**
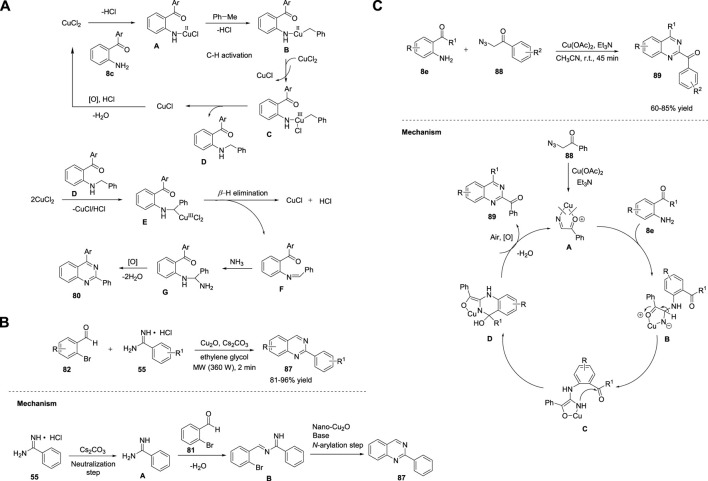
**(A)** Cu-catalyzed dual oxidative benzylic C-H amination for the synthesis of quinazolines **(B)** Cu2O nanocubes-catalyzed synthesis of quinazolines **(C)** Synthesis of quinazolines *via* Cu-catalyzed transimination.

In this reaction, a variety of electron-donating and electron-withdrawing groups are well tolerated, and, surprisingly, aliphatic substituents of 2-aminobenzoketones also give products in excellent yields. Cu acts as a dual catalyst in the construction of one C=N bond and one C-N bond in a single step. A plausible mechanism proposed by the authors suggests that the coordination of 2-aminobenzoketone **8c** with the Cu(II) species produces complex **A** *via* a ligand exchange reaction. Then, benzyl/Cu(II) species **B** is formed *via* benzylic C-H activation of complex **A**. Complex **B** then undergoes oxidation with another Cu(II) to give benzyl/Cu(III) complex **C** and one Cu(I) species. Next, reductive elimination of complex **C** generates intermediate **D** and another Cu(I) species. Simultaneously, **D** is converted into **F** *via* β-H elimination. In the last step, intermediates **F** and NH_3_ are converted to **G** *via* a similar catalytic cycle, which then forms **80** through condensation and oxidation. The Cu(I) species are then oxidized to the corresponding Cu(II) species in the presence of TBHP (Scheme 12A).

Ma *et al.* (2016) reported a novel three-component reaction for the synthesis of quinazolines **81** *via* Cu-catalyzed oxidative amination. Quinazolines were obtained in 33%–95% yields by employing *N*-alkylamide *viz.* 2-aminobenzophenone **8e** and DMSO as the sp^3^ carbon source, ammonium acetate **10** as the nitrogen source, O_2_ as the green oxidant, and CuCl as the catalyst in acetic acid at 110°C in air for 12 h (Scheme 11Cb) (Duan et al., 2016). Moreover, aromatic aldehydes and primary alcohols served as carbon sources to afford the corresponding 2-aryl and 2-alkyl quinazolines **81**. The approach is simple, atom-economical, and external oxidant-free, and DMSO acts as both a solvent and reagent.

Similarly, Wu *et al.* (2016) reported an efficient three-component reaction of 2-bromobenzaldehydes **82**, benzyl amines **33**, and sodium azide (N source) for assembling quinazolines **83** in 36%–82% yields *via* the Cu(I)/L-proline catalytic system in DMSO at 80°C for 15 h (Scheme 11Cc) (Xu et al., 2016). Except for the steric effect of 2-bromobenzaldehydes **82** on electron-deficient substrates, the remaining heteroaryl and electron-rich benzylamines **33** produced quinazolines **83** in good yields. *In situ*-generated aryl azides, readily available substrates, and dual activity of Cu(I) catalysts to promote S_N_Ar and denitrogenation/cyclization are remarkable features of this approach.

Adopting the same strategy, Chen et al. (2017) developed a Cu-catalyzed one-pot methodology for the synthesis of 4-aminoquinazolines **86**. The CuBr/L-proline-catalyzed reaction proceeded by the treatment of 2-iodo/2-bromobenzimidamides **84**, aldehydes **85**, and sodium azide in DMF at 70°C for 4 h to afford quinazolines **86** in 50%–90% yields (Scheme 11Cd) (Yang et al., 2017). The reaction mechanism involved consecutive S_N_Ar substitution, reduction, cyclization, oxidation, and tautomerization. Simple operation conditions, low temperatures, base-free reaction, and easy availability of starting precursors are the notable advantages of this approach.

In recent years, sonochemical techniques have received considerable attention for the preparation of inorganic nanomaterials such as monometallic, bimetallic, carbide, and oxide (Li et al., 2021). As an alternative to conventional methods, ultrasonication is considered favorable, cost-effective, powerful, eco-friendly, and employs easy reaction parameters that will increase the selectivity and yield of the product. Bhanage *et al.* (2017) reported the facile ultrasonic-assisted green synthesis of Cu_2_O-nanocubes. Furthermore, Cu_2_O nanocubes were used as heterogeneous nanocatalysts in the microwave-assisted preparation of quinazolines **87** at 360 W for 2 min *via* tandem cyclization of 2-bromobenzaldehydes **82** with amidines **55** in the presence of Cs_2_CO_3_ in ethylene glycol (Scheme 12B) (Raut et al., 2017).

A variety of electron-donating and electron-withdrawing substrates underwent the reaction smoothly and produced quinazolines **87** in 81%–96% yields. Ligand-free reaction, shorter reaction times, and recyclability of the catalyst at least four times are the merits of this green approach. The proposed mechanism begins with the neutralization of benzamidine hydrochloric salt **55** to give the amidine species **A**, which then react with 2-bromobenzaldehyde **82** to form imine intermediate **B**. Imine **B** undergoes Ullmann type *N*-arylation, generating the desired product, quinazoline **87** (Scheme 12B).

Kamal et al. (2017) developed an operationally simple approach for quick production of quinazoline derivatives *via* a Cu-catalyzed base-mediated system. The method involved the transimination of *o*-aminoketones **8** with *in situ*-generated imine, obtained from the decomposition of phenacyl azides **88,** in the presence of Cu(OAc)_2_ in Et_3_N as the base and acetonitrile as the solvent, at room temperature for 45 min, enabling the formation of substituted quinazolines **89** in 60%–85% yields (Scheme 12C). (Sastry et al., 2017) The reaction proceeded smoothly with a wide range of substituents on the aromatic ring. However, alkyl acyl azide instead of **88** failed to form the desired product. Mechanistically, the Cu(OAc)_2_/Et_3_N catalytic system initiates the loss of nitrogen from phenacyl azide **88** and generates Cu-chelated imine **A**. Transimination between *o*-carbonyl aniline **8e** and imine results in intermediate **B**. Deprotonation of **B** provided intermediate **C**. Nucleophilic attack of the amine group of intermediate **C** on the carbonyl group gives **D**. Finally **D**, undergoes aromatization in the presence of atmospheric oxygen to afford quinazoline **89** (Scheme 12C).

**SCHEME 13:**
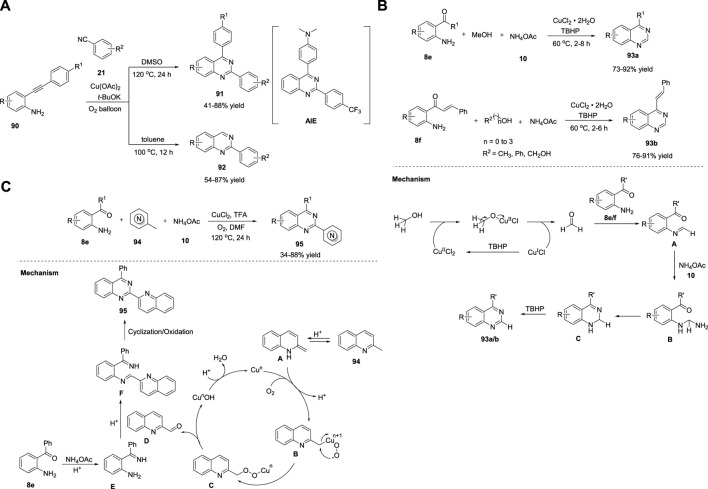
**(A)** Cu(OAc)_2_ catalyzed C-C bond cleavage for the synthesis of quinazolines **(B)** Cu-catalyzed oxidative amination of methanol for the synthesis of quinazolines **(C)** Cu-catalyzed aerobic oxidative cyclization for the synthesis of quinazolines.

Jiang *et al.* (2018) demonstrated the concise construction of substituted quinazolines **91**–**92** *via* Cu(OAc)_2_-catalyzed cyclization of 2-ethynylanilines **90** with benzonitriles **21** in the presence of *t*-BuOK as a base, toluene/DMSO, and molecular O_2_ at 100°C–120°C for 12–24 h (Scheme 13A). (Wang et al., 2018) This strategy enabled the effective assembly of C-N and C-C bonds and aerobic C-C triple bond cleavage. The major advantages of this strategy are readily available starting materials and catalysts, broad functional group tolerance with 41%–88% yields, and no external oxidant. However, heterocyclic substituted alkynes fail to afford the desired product. Furthermore, a new solid-state blue-emitting organic molecule was identified during the procedure, which has drawn attention for further development of a new variety of aggregation-induced emission (AIE) luminescent materials.

Ilangovan et al. (2019) illustrated a straightforward method for the assembly of quinazolines **93** using naturally abundant methanol as the C1 carbon source. The one-pot reaction of 2-aminoarylketones **8e** or 2-amino chalcone **8f**, alcohols, and ammonium acetate **10** in the presence of CuCl_2_·2H_2_O in TBHP at 60°C for 2–8 h led to the formation of quinazolines and styryl quinazolines **93a/b** in 73%–92% yields (Scheme 13B). (Satish et al., 2019) Atom-conomy, easy scale-up to gram quantities, methanol as a carbon source, and high yields are the benefits of this protocol. The plausible mechanism depicted in Scheme 13B begins with the dehydrogenation of methanol to produce formaldehyde in the presence of CuCl_2_·2H_2_O/TBHP. Subsequently, the *in situ*-generated formaldehyde is condensed with the aromatic primary amine of **8e/f** to produce imine species **A**. Intermediate **A** reacts with ammonium acetate **10** to produce aminal **B**, which then undergoes intramolecular cyclization and oxidation to produce the desired quinazoline **93a/b**.

As an improvement, Tang *et al.* (2019) reported a one-pot method to produce 2-azaaryl quinazolines **95** from 2-aminophenyl ketones **8e**, methylazaarenes **94**, and ammonium acetate **10** using CuCl_2_ as the catalyst and trifluoroacetic acid in DMF at 120°C for 24 h (Scheme 13C). (Liang et al., 2019) This aerobic oxidative cyclization provided several substituted quinazolines in 34%–88% yields. The features of this method include easily available starting precursors, mild reaction conditions, green oxidants, and good substrate applicability. The tentative mechanism proposed by the authors suggests the isomerization of **94** with acid-generated enamine intermediate **A**. Next, **A** is combined with Cu salts and O_2_ to give intermediate **B**, which then undergoes intramolecular rearrangement to form intermediate **C**, leaving behind quinoline-2-carbaldehyde **D**. Intermediate **E** formed from **8e** reacts with quinoline-2-carbaldehyde **D** to produce imine intermediate **F** through acid-catalyzed dehydration. In the last step, imine intermediate **F** undergoes cyclization and oxidation to afford desired product **95** (Scheme 13C).

**SCHEME 14:**
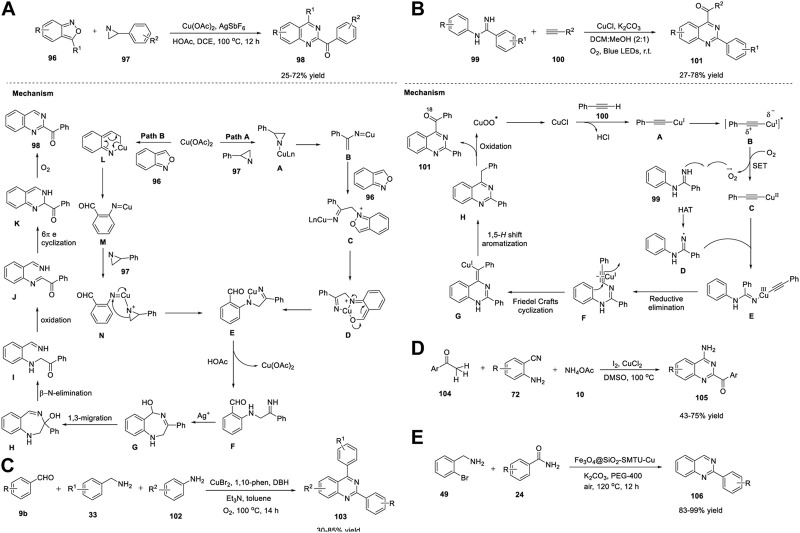
**(A)** Cu/Ag-catalyzed synthesis of quinazolines **(B)** Cu-catalyzed photoredox synthesis of quinazolines **(C)** Cu-catalyzed [3 + 2 + 1] annulation for the synthesis of quinazolines **(D)** I_2_/CuCl_2_-copromted [4 + 1 + 1] cyclization for the synthesis of quinazolines **(E)** Fe_3_O_4_@SiO_2_-SMTU-Cu nanocomposite catalyzed synthesis of quinazolines.

Xie et al. (2020) reported an unprecedented approach for the synthesis of quinazoline derivatives **98** by employing Cu/Ag-catalyzed annulation of anthranils **96** with azirines **97**. This novel strategy involving the cleavage of the C-N bonds of 2H-azirines attacked by anthranils as nucleophiles has not been explored before. The fact that 2H-azirines can act as nucleophiles to cleave the N-O bond of anthranils may be the reason. The optimized conditions included the reaction of anthranils **96** with phenyl-2H-azirine **97** in the presence of Cu(OAc)_2_ as the catalyst, AgSbF_6_ as the additive, acetic acid in DCE, and O_2_ at 100°C for 12 h to produce (quinazolinyl-2-yl)methanones **98** in 25%–72% yields *via* an unexplored 1,3-hydroxyl migration and β-N elimination (Scheme 14A) (Sun et al., 2020).

Steric hindrance did not influence the reaction by accommodating a variety of electronically and sterically variable functional groups, and the product was produced in good yields. However, 3-(thiophene-2-yl)-2H-azirine **97** did not afford the desired product, which is a minor limitation in the substrate scope. Atom-economy, easy scale-up with 70% yield, cheap and abundant catalysts, and good substrate compatibility are the benefits of this protocol. Mechanistically, azirine **97** coordinates with the Cu catalyst to generate complex **A**. Azirine complex **A** then transforms into the corresponding nitrene–Cu complex **B** *via* reversible N-C (Jafari et al., 2016) bond cleavage. Anthranil **96** attacks complex **B** to give intermediate **C**. Cu is then inserted into the adjacent N-O bond of **C** to produce intermediate **D**, which then undergoes isomerization to form complex **E** (path A). Alternatively, a Cu salt can be inserted into the cleavable N−O bond of anthranil **96**, resulting in Cu–nitrenoid species **M**, which further coordinates with 2H-azirine **97** to afford intermediate **N**. Subsequently, migratory insertion of nitrenoid species into the N-C (Jafari et al., 2016) bond in **N** provides complex **E** (path B). **E** is protonated by HOAc and generates intermediate **F**, leaving behind Cu(II) acetate by closing the catalytic cycle. AgSbF_6_ promotes the cyclization of **F** to deliver intermediate **G**. Subsequently, 1,3 migration of the hydroxy group and β-N elimination gives α-aminocarbonyl intermediate **I**, which then undergoes coordination with Cu(II) and oxidizes to form intermediate **J**. Intermediate **J** then transforms into 1,2-dihydroquinazoline product **K** *via* 6π electrocyclization. In the last step, autoxidation of **K** affords quinazoline **98** (Scheme 14A).

Traditional photocatalytic reactions mostly demand expensive transition metals, specially designed ligands, organic oxidants, and reaction waste. Visible-light photoredox catalysis (PRC) has emerged as a complementary approach to traditional photocatalysis. Hwang *et al.* (2021) achieved visible-light-induced photoredox Cu-catalyzed oxidative Csp (Jafari et al., 2016)-H annulation (Friedel–Crafts-type cyclization) of amidines **99** with terminal alkynes **100** to assemble functionalized quinazolines **101**. The oxidative Csp (Jafari et al., 2016)-H annulation of *N*-phenylbenzimidamide **99** with phenylacetylene **100** catalyzed by CuCl, by employing K_2_CO_3_ as a base, in DCM:MeOH (2:1) in presence of O_2_ at room temperature for 22–24 h produced quinazolines **101** in 27%–78% yields (Scheme 14B). (Charpe et al., 2021) The promising features of this approach include: a) the construction of 2,4-disubstituted quinazolines at RT through simultaneous C-C and C-N bond formation; b) use of cost-effective CuCl catalyst and O_2_ as an oxidant under mild reaction conditions; c) ligand-free; d) ease of scale-up; e) water as the only by-product; f) broad substrate scope; and g) green metrics and Eco-Scale evaluations proved that this photochemical process is simple and environmentally benign. The annulation mechanism begins with the absorption of blue light by the *in situ*-generated Cu(I)-phenylacetylide **A** (λ_max_ = 472 nm) with visible-light irradiation to form the photochemically excited triplet state **B** (long-lived triplet lifetime, *τ* = 15.95 μs). **B** then donates an electron to molecular O_2_ and generates Cu(II) complex **C** and superoxide anion radical *via* the SET process. Subsequently, owing to the basic nature of the copper superoxo radical anion, it abstracts acidic NH protons to generate nitrogen-centered radical **D**, which then reacts with Cu(II)–phenylacetylide complex **C** and delivers Cu(III)-complex species **E**. Upon reductive elimination, intermediate **E** gives the Cu(I)-coordinated ynamine intermediate **F**. Furthermore, intermediate **F** undergoes Friedel–Crafts-type cyclization (6-exo-dig cyclization) to form cyclized intermediate **G** and subsequent aromatization to form compound **H**, which then undergoes photo-oxidation by Cu(II) superoxo (λ_abs_ = 486 nm) to yield the desired product **101** (Scheme 14B).

Recently, Liu et al. (2021) reported a Cu-catalyzed multicomponent [3 + 2+1] annulation reaction of benzaldehyde **9b**, benzylamine **33**, and aniline **102** for the facile access to quinazolines **103**. The cascade reaction proceeded with the treatment of benzaldehyde **9**, benzylamine **33**, and aniline **102** in the presence of CuBr_2_ and 1,10-phenanthroline in dibromohydantoin (DBH) with triethylamine and toluene in O_2_ at 100°C for 14 h to produce quinazolines **103** in 30%–85% yields (Scheme 14C). (Wang et al., 2021) The broad substrate scope, ease of scale-up to 1.72 g with 68% yield, and oxygen as a green oxidant are the advantages of this protocol. This is the first analogous three-component synthesis that employs benzaldehyde, benzylamine, and aniline for the assembly of quinazoline derivatives.

In the same year, Wu et al. (2021) demonstrated that I_2_/CuCl_2_-copromoted diamination of C(sp^3^)-H bonds for the facile synthesis of quinazolines **105**. The [4 + 1+1] annulation reaction between methyl ketones **104**, 2-aminobenzonitriles **72**, and ammonium acetate **10** in the presence of I_2_/CuCl_2_ in DMSO at 100°C resulted in the formation of 2-acyl-4-aminoquinazolines **105** in good yield (Scheme 14D). (Huang et al., 2021) This became the first example of the assembly of 2-acyl-4-aminoquinazolines using a methyl group as an input. Multiple C-N bond formation, dual C(sp^3^)-H amination, broad functional group compatibility, and multicomponent cyclization are the features of this approach.

Recently, Sajjadi *et al.* (2022) reported that the Fe_3_O_4_@SiO_2_–SMTU–Cu nanocomposite catalyzed the synthesis of quinazolines **106** from 2-bromobenzylamine **49** and benzamides **24** in K_2_CO_3_ and PEG-400 in air at 120°C for 12 h (Scheme 14E). (Riadi et al., 2022) The reaction proceeded smoothly with a variety of functional groups and delivered product **106** in 83%–99% yields. This is the first report of the synthesis of quinazolines using a magnetic copper nanocatalyst. The catalyst was fabricated through immobilization of Cu(NO_3_)_2_ on the surface of silica-coated magnetic Fe_3_O_4_ NPs (Fe_3_O_4_@SiO_2_) functionalized with the *S*-benzylisothiourea ligand. The notable merits of this strategy include excellent yields, easy catalyst separation using an external magnet, shorter reaction times, non-toxic Cu catalyst, and simple operation procedure.

## 3 Second-row transition metal catalysts

### 3.1 Ruthenium

Ruthenium, a rare transition metal, has multiple oxidation states. As compared to other precious transition metals, it is less expensive and acts as an important catalyst for synthetic transformations, such as hydrogenation reactions (Sayed et al., 2020), oxidative transformations (Dutta et al., 2019), and coupling reactions (Duarah et al., 2018).

**SCHEME 15:**
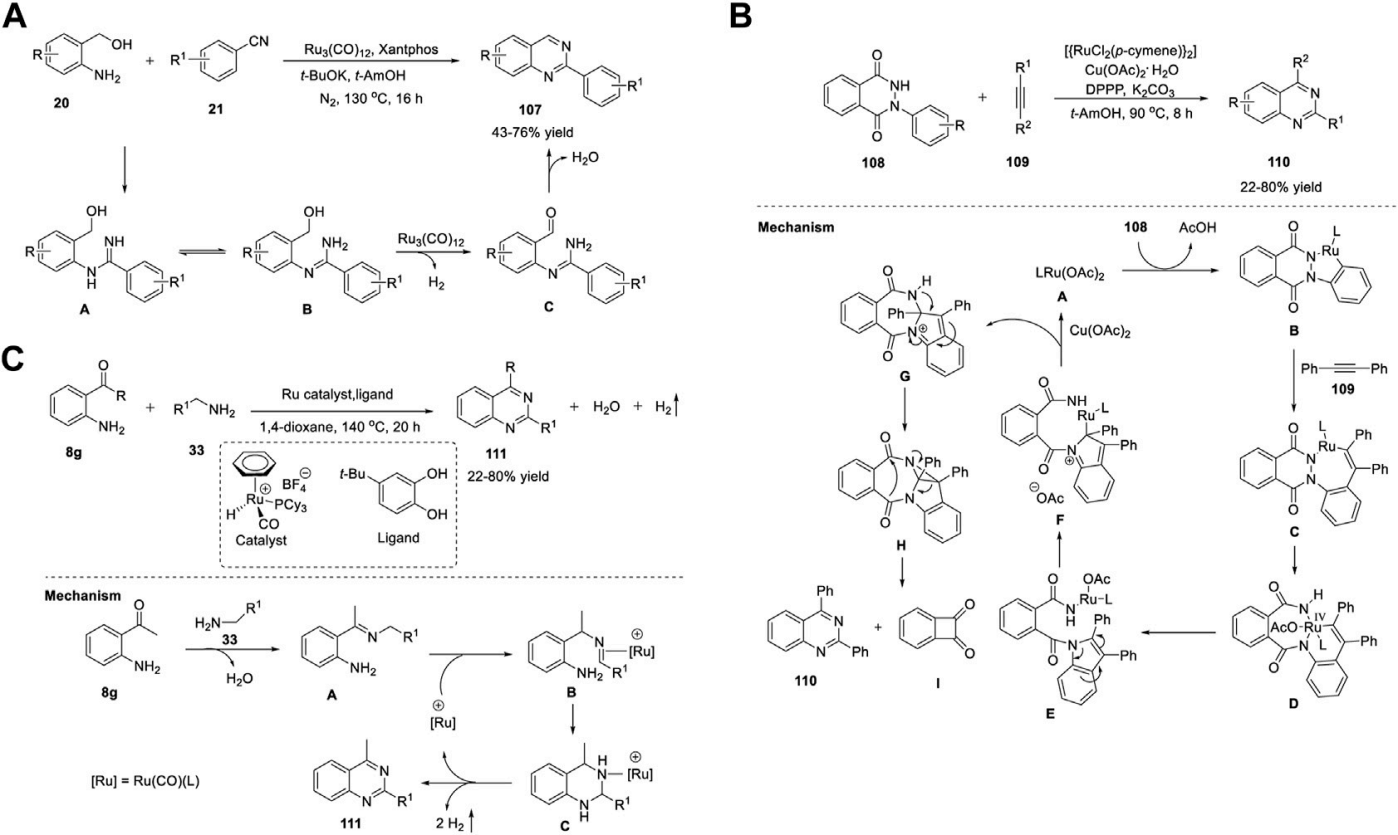
**(A)** Ru_3_(CO)_12_ catalyzed ADC coupling for the synthesis of quinazolines **(B)** Ru-catalyzed C-H activation and annulation for the synthesis of quinazolines **(C)** Ligand-promoted Ru-catalyzed synthesis of quinazolines.

Zhang et al. (2014) reported a Ru_3_(CO)_12_/Xantphos/*t*-BuOK catalyst system for the ADC reaction that yields 2-arylquinazolines **107**. Treatment of 2-aminoaryl methanols **20** with benzonitriles **21** in the presence of Ru_3_(CO)_12_ as the catalyst, Xantphos as the ligand, and *t*-BuOK as the base in *t*-AmOH in N_2_ at 130°C for 16 h produced 2-arylquinazolines **107** in 43%–76% yields (Scheme 15A) (Chen et al., 2014a). Moreover, aliphatic nitriles **21** afforded the expected product in a lower yield (18% yield). Advantages of this protocol are its simple operation, broad substrate scope, high atom efficiency, and commercially available catalyst system. The ADC proceeds in the following sequence: 1) nucleophilic addition of the amino group of **20** to benzonitrile **21** generates an amidine intermediate **A**. 2) Next, the ruthenium-catalyzed dehydrogenation of the alcohol group of **A** yields *o*-carbonyl amidine **B**. 3) In the last step, intramolecular condensation of **B** produces quinazoline **107** (Scheme 15A).

Gogoi *et al.* (2018) developed an unprecedented Ru(II)-catalyzed C-H activation and annulation approach for the facile access to quinazolines **110** *via* C-C triple bond cleavage. The reaction of 2-phenyldihydrophthalazinedione **108** with alkyne **109** using [{RuCl_2_(*p*-cymene)}_2_] as the catalyst and Cu(OAc)_2_·H_2_O as the additive, 1,3-bis-(diphenylphosphino)propane (DPPP) as the ligand, and K_2_CO_3_ as the base in *t*-AmOH at 90°C for 8 h delivered quinazolines **110** in 22%–80% yields (Scheme 15B). (Prakash et al., 2018) In this cleavage reaction, both fragments of the alkyne remain in the product. Both the partners *viz. N*-arylpyrazol-5-ones and diaryl-/arylalkyl-substituted alkynes are well tolerated with good yields. However, dialkyl-, terminal-, silyl-, and bromo-substituted alkynes did not favor the reaction. The reaction mechanism is initiated by the active Ru(II) catalyst **A** generated from [RuCl_2_(*p*-cymene)_2_] and Cu(OAc)_2_. The active catalyst irreversibly reacts with **108** to produce complex **B** through the activation of the C-H bond. The next step is the insertion of alkyne **109** into the C-Ru bond to deliver a seven-membered Ru(II) complex **C**. Oxidation of **C** leads to the formation of Ru(IV) complex **D** *via* cleavage of the N-N bond. Subsequent reductive elimination–activation at C-2 reductive elimination–intramolecular cyclization–intramolecular rearrangement and elimination generates the indole derivative **E**, Ru complex **F**, seven-membered amide **G**, diazacyclopropane azulene **H**, quinazoline derivative **110**, and diketo compound **I** in a stepwise manner (Scheme 15B).

Yi et al. (2019) reported a novel ligand-promoted Ru-catalyzed dehydrogenative coupling reaction for the facile access to quinazolines **111**. The coupling reaction between 2-aminophenyl ketones **8** and amines **33** in the presence of an *in situ*-generated catalytic system, Ru–hydride complex [(C_6_H_6_)(PCy_3_)(CO)RuH]^+^ BF_4_^−^, and 4-(1,1-dimethylethyl)-1,2- benzenediol (L1) as the ligand in 1,4-dioxane at 140°C for 20 h generated quinazoline derivatives **109** in 45%–87% yields (Scheme 15C). (Arachchige and Yi, 2019) The prominent advantages of this method are as follows: a) readily available substrates, b) wide substrate scope accommodating common functional groups with good yields, c) no need for any reactive reagents, and d) water and hydrogen are the only byproducts of the reaction. A plausible mechanism proposed by the authors suggests that the initial dehydrative coupling of amino ketone **8g** and amine **33** generates imine intermediate **A**. Then, the Ru catalyst facilitates imine **A** isomerization to give imine-coordinated species **B**, which upon cyclization and dehydrogenation furnishes the desired product **111**. The authors believe that the redox-active catechol ligand may facilitate the dehydrogenation step in catalysis (Scheme 15C).

**SCHEME 16:**
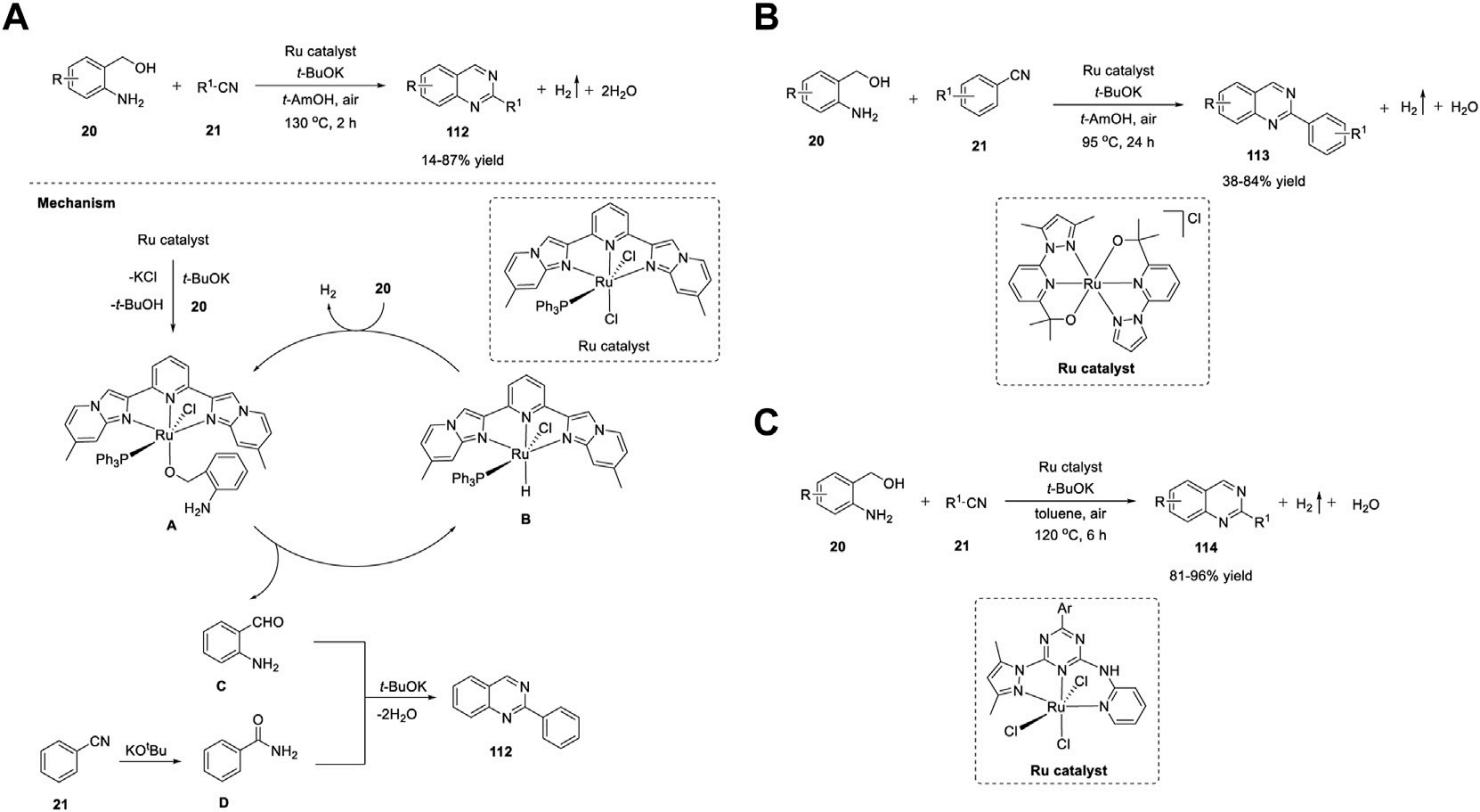
**(A)** Ru(II) *NNN* pincer catalyzed synthesis of quinazolines **(B)** Ru(III)-catalyzed ADC synthesis of quinazolines **(C)** Ru *NNN* pincer catalyzed acceptorless dehydrogenative synthesis of quinazolines.

Hao et al. (2019) first reported the synthesis of quinazolines **112** catalyzed by the *NNN* pincer Ru(II) complex *via* the ADC reaction of *o*-aminobenzyl alcohols **20** and (hetero)aryl or alkyl nitriles **21** in the presence of *t*-BuOK as the optimal base in *tert*-AmOH in air at 130°C for 2 h (Scheme 16A) (Wan et al., 2019). This strategy has gained attention because it overcomes the limitations of previously reported Ru-catalyzed syntheses and exhibits advantages such as shorter reaction time (2 h) compared with previous synthesis (16 h), compatibility with aliphatic nitriles, affording up to 87% yield (18%), and environmental benignity.

Mechanistically, in the presence of *t*-BuOK, *o*-aminobenzyl alcohol **20** reacts with the Ru catalyst to generate Ru-alkoxide **A**, which then undergoes β-elimination to produce Ru-H species **B** and carbonyl intermediate **C**. The reaction of Ru-H species **B** with **20** regenerates Ru-alkoxide intermediate **A** by evolving hydrogen gas. Simultaneously, the base promotes the hydration of benzonitrile **21**, followed by cyclocondensation to afford the desired quinazoline **112** (Scheme 16A).

Hao et al. (2021) developed an *N*,*N*,*O*-tridentate pyrazolyl-pyridinyl-alcohol ligand-supported Ru(III) complex for the facile synthesis of quinazolines **113**. The dehydrogenative coupling of 2-aminoaryl methanols **20** with nitriles **21** in the presence of Ru(III) complex L_2_RuCl and *t*-BuOK in *tert*-AmOH at 95°C for 24 h delivered product **113** in 38%–84% yields (Scheme 16B). (Huo et al., 2021) The easy preparation, air/moisture stable catalyst, atom economy, phosphine ligand-free, sustainable strategy, and broad functional group tolerance are noteworthy merits of this approach.

Recently, Das et al. (2022) demonstrated an efficient method for the Ru *NNN*-pincer complex-catalyzed synthesis of quinazoline derivatives **114** *via* ADC of 2-amino benzyl alcohols **20** and nitriles **21** in the presence of *t*-BuOK in toluene at 120°C for 6 h. The products were obtained in 81%–96% yields (Scheme 16C). (Bhattacharyya et al., 2022) Remarkably, this catalytic system displayed a promising high TON of 290000 for 2-phenylquinazoline **114**, which is the highest value reported so far for transition metal-based catalysts.

### 3.2 Rhodium

Rhodium is one of the rarest and most expensive metals. This noble metal favors a wide range of chemical transformations such as hydrogenation (Schiwek et al., 2020), hydroformylation (Alsalahi and Trzeciak, 2021), and C-H activation (Zhou et al., 2016), in the form of Rh(II) and Rh(III) complexes.

**SCHEME 17:**
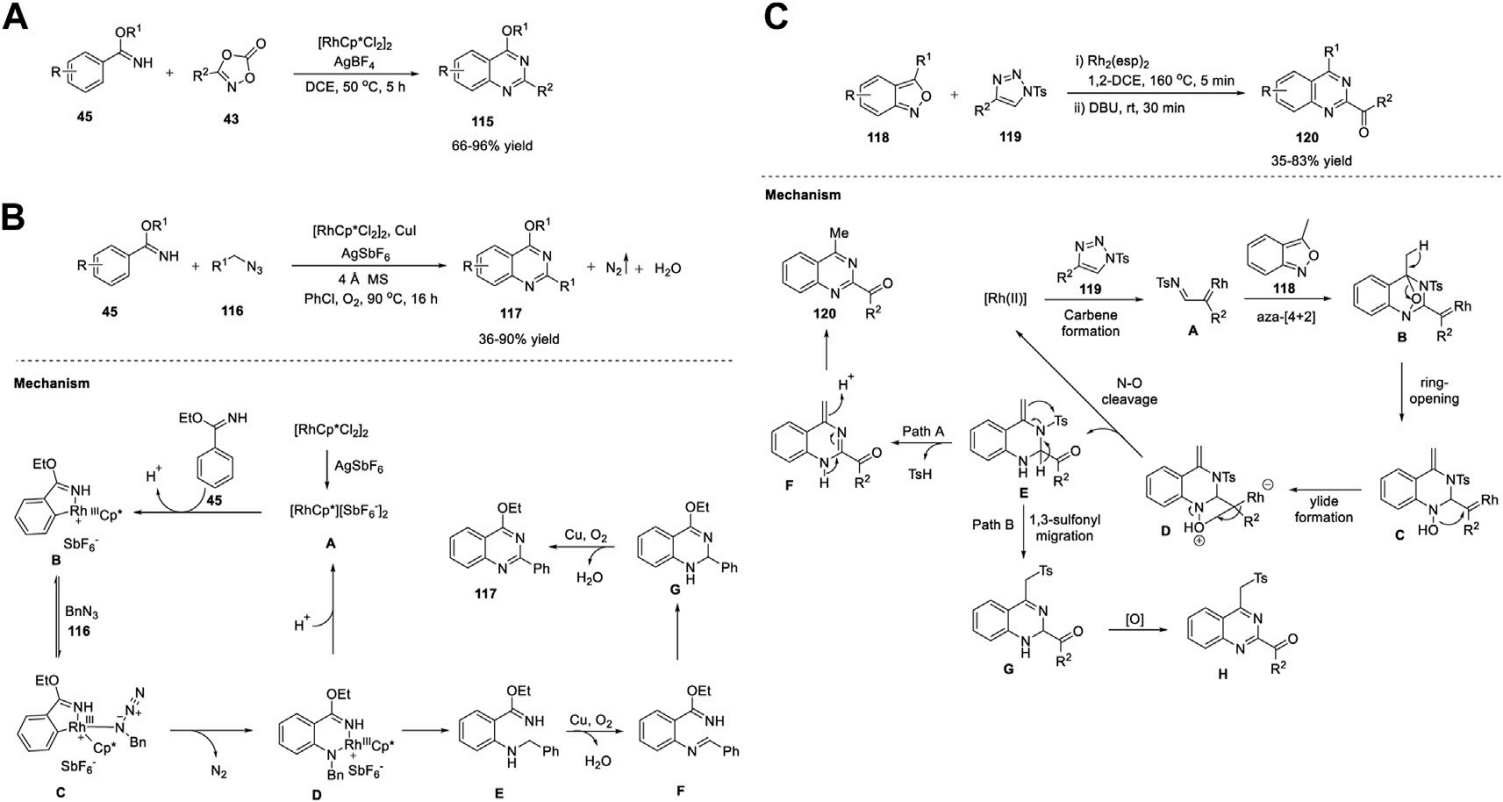
**(A)** Rh(III)-catalyzed synthesis of quinazolines **(B)** Rh- and Cu-co-catalyzed C-H activation for the synthesis of quinazolines **(C)** Rh(II)- catalyzed transannulation for the synthesis of quinazolines.

Transition-metal-catalyzed C-H functionalization has emerged as a shortened synthetic sequence in recent years for the construction of C-C/C-hetero bonds (Ye and Cramer, 2015). However, the presence of a nearby directing group is generally needed to direct the position of a metal catalyst for specific C-H bond activation. Zhu *et al.* (2016) reported a rarely explored double C−N bond formation strategy to construct highly substituted quinazolines **115** using benzimidates **45** and dioxazolones **43** in the presence of a redox-neutral [Cp*RhCl_2_]_2_/AgBF_4_ catalytic system with DCE as a solvent at 50°C for 5 h (Scheme 17A). (Wang et al., 2016c) A wide range of electron-donating and electron-withdrawing substrates perform well to afford the corresponding products in 66%–96% yields. Ideally, dioxazolone acts as an internal oxidant to maintain the catalytic cycle and a coupling partner to yield quinazolines **115**. The mechanism is similar to that presented for Co-catalyzed synthesis proposed by Li et al. (2016).

Jiao et al. (2016) presented a novel Rh- and Cu-co-catalyzed C-H bond activation and annulation approach for the facile access to quinazoline derivatives **117**. The aerobic oxidative [4 + 2] C-H annulation of imidate esters **45** and alkyl azides **116** in the presence of [Cp*RhCl_2_]_2_ as the catalyst, Cu(I) as the co-catalyst, AgSbF_6_ as the additive in 4 Å MS, and chlorobenzene under O_2_ at 90°C for 16 h furnished desired product **117** in 36%–90% yields (Scheme 17B). (Wang and Jiao, 2016) This is the first report of [4 + 2] C-H annulation with carbon–heteroatom synthons. Atom economy, O_2_ as a green oxidant, N_2_ and H_2_O as the only byproducts, broad substrate scope, and the formation of functionalized quinazoline are the benefits of this approach. Mechanistically, the reaction proceeds *via* initial cationic Cp*Rh(III) catalyst **A** formation with the help of AgSbF_6_. Subsequently, imidate group **45** directs C-H bond activation, generating rhodacyclic intermediate **B**, which can coordinate with benzyl azide **116** to give intermediate **C**. Subsequently, **C** undergoes migratory insertion to furnish Rh species. Protonation of complex **D** regenerates cationic Cp*Rh(III) **A** and produces intermediate **E** *via* amination. The amine undergoes Cu-catalyzed aerobic oxidation to produce the corresponding imine **F** with the removal of H_2_O as a byproduct. In the last step, cascade intramolecular addition and aerobic oxidative aromatization lead to the formation of quinazoline **117** (Scheme 17B).

Rh-azavinylcarbene (Rh-AVC) has evolved as a versatile intermediate for the formation of *N*-heterocycles (Davies and Alford, 2014). Rh-AVC can be readily generated from *N*-sulfonyl-1,2,3-triazoles *via* denitrogenation catalyzed by the Rh(II) system and is widely used as a [1C]-, [2C]-, and aza-[3C]-component in several transformations (Miura et al., 2012; Lei et al., 2015; Ryu et al., 2015). Interestingly, Tang *et al.* (2016) reported an unprecedented Rh(II)-catalyzed transannulation of 2,1-benzisoxazoles **118** with *N*-sulfonyl-1,2,3-triazoles **119**. The cycloaddition of 2,1-benzisoxazoles **118** with *N*-sulfonyl-1,2,3-triazoles **119** catalyzed by Rh_2_ (esp)_2_ in 1,2-DCE at 160°C for 5 min and then with DBU at room temperature for 30 min delivered quinazolines **120** in 35%–83% yields (Scheme 17C). (Lei et al., 2016) Moreover, *N*-sulfonyl-1,2,3-triazole **119** acts as an aza-[2C]-component in this cycloaddition reaction, which represents the first example of using Rh(II)-AVC as an aza-[2C]-component in the relevant cycloadditions. However, 2,1-benzisoxazoles **118** with an H atom or phenyl group at the C-3 position failed to form the desired products, which limits the substrate scope; otherwise, it is a remarkable approach. The reaction mechanism (Scheme 17C) proposed by the authors suggests that the initial treatment of triazole **119** with Rh(II)- catalyst yields Rh-azavinylcarbene **A,** which then reacts with 2,1-benzisoxazole **118** to generate intermediate **B** *via* aza-[4 + 2] cycloaddition. Next, ring opening of **B** produces **C,** which then leads to oxonium ylide **D**. Subsequently, cleavage of the N-O bond results in intermediate **E** with the release of the Rh(II)-catalyst. At this stage, **E** can be further proceeded by two pathways. In one pathway, it can be converted to quinazoline **120** *via* elimination of TsH, followed by tautomerization. This process plays a key role in the presence of a DBU (path A). However, **E** could also undergo formal 1,3-sulfonyl migration (Xin et al., 2015) to generate intermediate **G**, which upon autoxidation with air delivers quinazoline derivative **H** (path B). However, concerted intramolecular 1,3-sulfonyl migration cannot be completely excluded. Non-etheless, a stepwise mechanism *via* a close ion-pair intermediate is more likely to account for the transformation from **E** to **120** based on crossover experiments.

**SCHEME 18:**
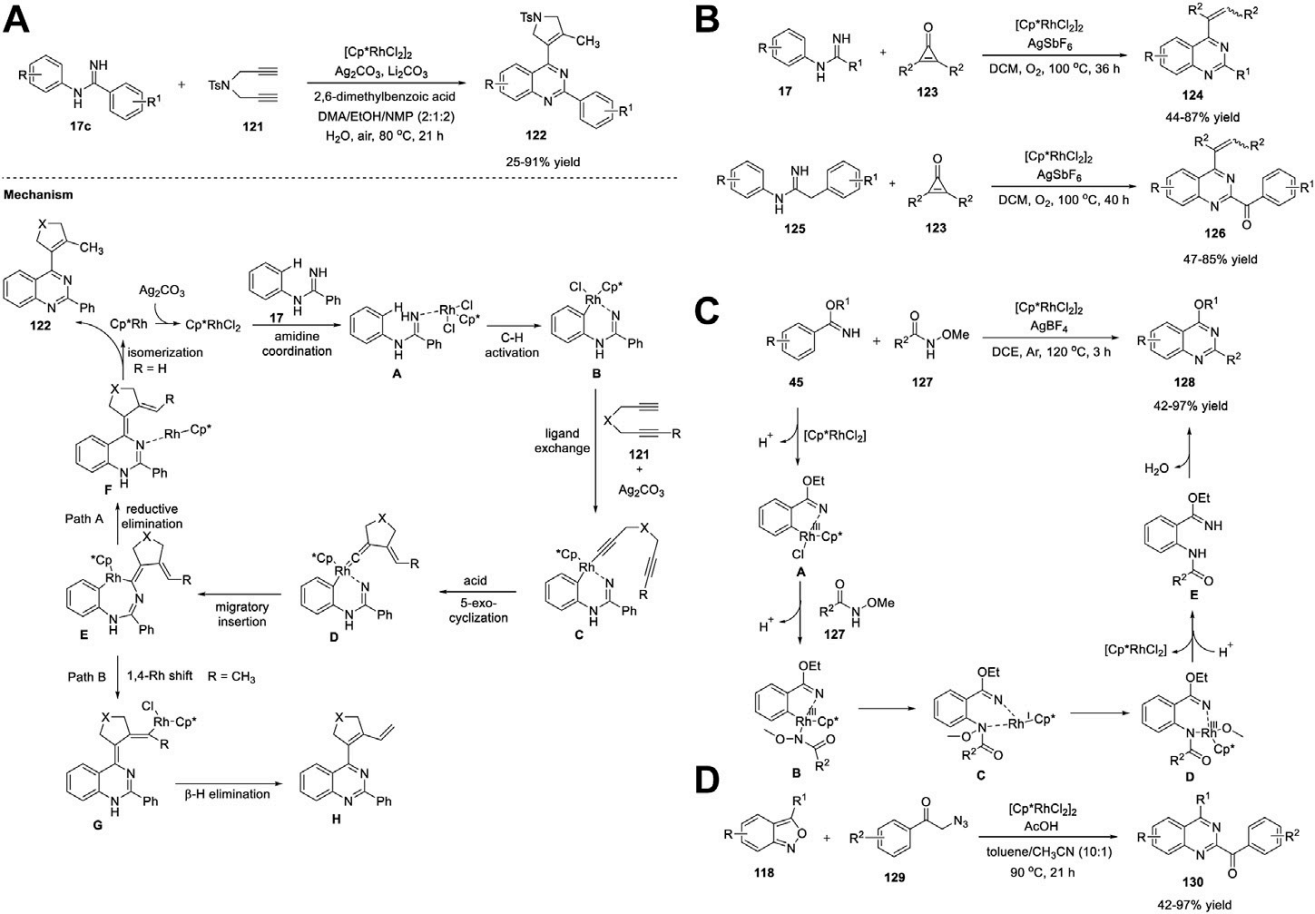
**(A)** Rh(III)-catalyzed [5 + 1] annulation for the synthesis of quinazolines **(B)** Rh(III)-catalyzed synthesis of quinazolines **(C)** Rh(III)-catalyzed one-pot cascade synthesis of quinazolines **(D)** Rh(III)-catalyzed tandem access to quinazolines.

Xu et al. (2018) demonstrated a rarely explored Rh(III)-catalyzed [5 + 1] annulation *via* direct C-H activation. The 5-exo-cyclization reaction of amidines **17c** with diynes **121** in the presence of [Cp*RhCl_2_]_2_, Ag_2_CO_3_, 2,6-dimethylbenzoic acid, H_2_O, Li_2_CO_3_, and DMA/EtOH/NMP (2:1:2) in air at 80°C for 21 h resulted in the formation of quinazolines **122** in 25%–91% yields (Scheme 18A) (Xu et al., 2018).

Notable features of this strategy are as follows: a) diyne was used for the first time as a one-carbon precursor in C-H activation, b) amidines and diynes display high reactivity in air and water, c) broad functional group tolerance, and d) two heterocyclic rings are assembled in one step by cascade C-H activation/[5 + 1] annulation.

The reaction mechanism begins with the initial formation of Rh(III)-complex **A**. Next, C-H activation at the *ortho*-H of the amidine bond followed by ligand exchange generates rhodacycle **C**. The 5-exo-cyclization of **C** results in intermediate **D**, which upon migratory insertion yields intermediate **E**. Furthermore, **E** displayed two distinct pathways. In Path A, reductive elimination of **E** gives intermediate **F** with liberation of Cp*Rh(I), which then undergoes reoxidization into the Cp*Rh(III) catalyst by Ag_2_CO_3_ (Wang et al., 2017). Subsequently, demetalation followed by isomerization affords the [5 + 1] product, quinazoline **122** (Path A) (Yang et al., 2016). In Path B (Xu et al., 2017), for unsymmetrical diynes bearing a methyl group at one alkyne terminus, step-wise migratory insertion-1,4-rhodium shift-β-H elimination produces the dehydrogenation product **H**, as evidenced by NMR (Scheme 18A).

Adopting the [5 + 1] annulation strategy, Wu *et al.* (2020) demonstrated an unprecedented formation of 4-alkene quinazolines using a Rh(III) catalyst. In recent years, cyclopropenones have been widely used as appealing reaction partners, with ring-strain release as the driving force.

C-H activation between *N*-arylamidine and cyclopropenone catalyzed by [Cp*RhCl_2_]_2_ with AgSbF_6_ in DCM in O_2_ at 100°C for 36 h led to the construction of 4- ethenyl quinazolines in 44%–87% yields (Scheme 18B). (Xing et al., 2020) Moreover, 2-benzoyl quinazolines were obtained in good yields from *C*-benzyl imidamides *via* an additional oxidation step. Mild reaction conditions, high atom economy, and good substrate compatibility are merits of this protocol.

In the same year, Dong *et al.* (2020) took a step ahead and employed *N*-methoxyamide as an amidating agent for the first time, instead of dioxazolones or alky azides. The one-pot cascade reaction proceeded by the treatment of imidate esters with *N*-alkoxyamides utilizing [Cp*RhCl_2_]_2_ with AgBF_4_ in DCE in Ar at 120°C for 3 h, led to the production of quinazolines in 42%–97% yields (Scheme 18C). (Xu et al., 2020) Furthermore, under the standard reaction conditions, a series of *N*-methoxyamides, including heteroarylamides and 2-napthamides, gave the desired product in good yields. Moreover, the challenging aminating reagents from cinnamic acid derivatives also furnished the product, which could not be obtained using previously reported methods. The Rh(III)-catalyzed [4 + 2] reaction proceeds *via* C−H activation/amidation/annulation in a step-wise manner.

Very recently, Yu et al. (2022) reported that a novel Rh(III)-catalyzed tandem reaction of 2,1-benzisoxazoles and α-azido ketones in the presence of [Cp*RhCl_2_]_2_ as the catalyst, AcOH as the additive, and toluene/acetonitrile (10:1) as the solvent system at 90°C for 21 h delivered (quinazolin-2-yl)methanone derivatives in 21%–88% yields (Scheme 18D). (Liu et al., 2022) This tandem conversion involves Rh(III)-catalyzed denitrogenation of α-azido ketones, aza-[4 + 2] cycloaddition, ring opening, and dehydration aromatization. The key step in the synthesis is the aza-[4 + 2] cycloaddition of the imine Rh-complex with 2,1-benzisoxazole.

### 3.3 Palladium

Palladium is a non-abundant precious metal that displays variable oxidation states, high stability, and excellent selectivity. It can facilitate some exclusive transformations, such as cross-coupling reactions (Zhou et al., 2019) and hydrogenation (Reddy and Swamy, 2017).

**SCHEME 19:**
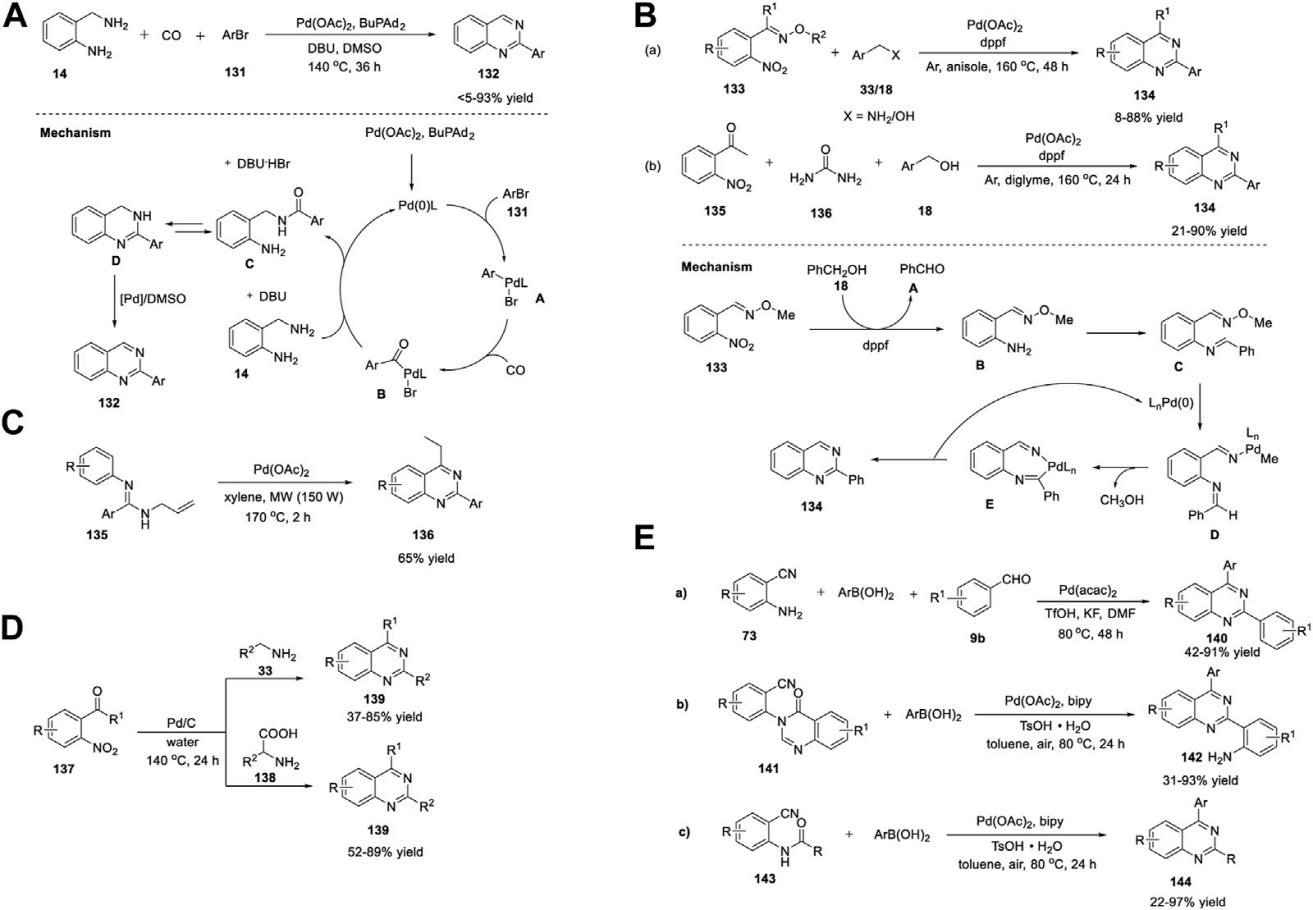
**(A)** Pd-catalyzed carbonylative synthesis of quinazolines **(B)** Pd-catalyzed carbonylative synthesis of quinazolines **(C)** Pd-catalyzed synthesis of quinazolines *via* hydrogen transfer strategy **(D)** Pd-catalyzed synthesis of quinazolines under microwave irradiation **(E)** Heterogenous Pd-catalyzed synthesis of quinazolines *via* HT approach.

Wu et al. (2014) reported the first carbonylative approach towards quinazoline formation from easily available 2-aminobenzylamine and aryl bromides. The carbonylative coupling of 2-aminobenzylamine **14** with aryl bromides **131** using Pd(OAc)_2_ and BuPAd_2_ with DBU as the base in DMSO at 140°C for 36 h generated quinazoline **132** scaffolds in <5%–93% yields (Scheme 19A). (Chen et al., 2014b) DMSO served both as a solvent and oxidant in this reaction. The one-pot reaction proceeded in a sequential manner, that is, aminocarbonylation, condensation, and oxidation. The substrate scope was extended to include several electron-donating and electron-withdrawing groups. Particularly, good to moderate yields were achieved, with the exception of multifluoro-substituted bromobenzene and more steric 2,4,6-trimethylbromobenzene, both of which failed to give the desired product.

The plausible mechanism depicted in Scheme 19A suggests that the reduced Pd(0) commenced the catalyst cycle, followed by the oxidative addition of aryl bromides **131** to Pd(0), resulting in the corresponding organopalladium species **A**. Subsequent coordination and insertion of CO delivered the key acylpalladium complex **B**. Nucleophilic attack of **14** intermediate **B** led to compound **C** and Pd(0) with the aid of a base. Compound **C** then underwent intramolecular cyclization to generate intermediate **D**, which was then oxidized to the terminal quinazoline product **132** in the presence of Pd and DMSO.

In the same year, another one-pot method was presented by Deng et al. (2014) for the efficient assembly of 2-aryl quinazolines using a Pd catalytic system for the first time (Scheme 19B). (Wang et al., 2014) This hydrogen transfer strategy proceeded *via* the reaction of a) (*E*)-2-nitrobenzaldehyde *O*-methyl oximes **133** and alcohols **18** or benzyl amines **33** or of b) 1-(2-nitrophenyl)ethenone **135**, urea **136**, and benzyl alcohols **18** under the optimized conditions of Pd(OAc)_2_ and DPPF (1,1′-Bis(diphenylphosphino)ferrocene) ligand in anisole/diglyme at 160°C for 24–48 h and produced 2-aryl quinazolines in 8%–90% yields (Scheme 19B). In this protocol, the nitro group was reduced by the hydrogen evolved from the alcohol dehydrogenation step. Mechanistically, benzaldehyde **A** is generated by the dehydrogenation of benzyl alcohol **18** and reduction of **133** to form intermediate **B**, which then undergoes condensation to as by-products. In the last step, the reductive elimination of **E** gave imine intermediate **C**. Oxidative addition, followed by palladation and cyclization, afford intermediate **E** and methanol results in quinazoline **134,** and the active Pd(0) catalyst is released (Scheme 19B).

Wang et al. (2015) described Pd-catalyzed C-H activation under microwave irradiation for the synthesis of quinazolines. *N*-allylamidines **135** underwent C-H activation in the presence of Pd(OAc)_2_ in xylene under microwave irradiation (150 W) at 170°C for 2 h, resulting in quinazolines **136** in 65% yield and a mixture of quinazoline and imidazole in 73%–94% yields (Scheme 19C) (Xu et al., 2015), and the latter could be separated easily through column chromatography.

Tang et al. (2016) proposed another hydrogen transfer protocol utilizing a heterogeneous Pd-catalytic system for the efficient synthesis of 2,4-disubstituted quinazolines *via* the direct oxidative amination of C (sp^3^)-H bonds with nitro groups, which has not been previously reported. The first example of the reaction of *o*-nitroacetophenones with benzyl amines or amino acids catalyzed by Pd/C in water at 140°C for 24 h produced quinazolines in 37%–89% yields (Scheme 19D). (Tang et al., 2016) The reaction proceeded in a C-N bond cleavage formation sequence. Furthermore, when amino acids were used as substrates, the product was synthesized *via* decarboxylative oxidative amination. The recyclability of the catalyst for at least five times; broad substrate scope; tolerance to air; oxidant-, ligand-, and base-free; and usage of water as the solvent make this protocol remarkable.

Chen et al. (2018) reported the synthesis of quinazolines using three different methodologies: a) the one-pot reaction of 2-aminobenzonitriles, aldehydes, and arylboronic acids employing Pd (acac)_2_ as the catalyst, 5,5′-dimethyl-2,2′-bipyridine as the ligand, TfOH as the additive, and DMF as the solvent in KF at 80°C for 48 h produced quinazolines in 42%–91% yields (Scheme 19Ea) (Hu et al., 2018). This is the first report of one-pot, three-component tandem quinazoline synthesis *via* carbopalladation of the cyano group. b) The tandem sequential nucleophilic addition and intramolecular cyclization of readily available 2-(quinazolinone-3(4H)-yl)benzonitriles with arylboronic acids led to the preparation of quinazoline derivatives in 31%–93% yields in the presence of Pd(OAc)_2_, bipyridine, and TsOH·H_2_O in toluene in air at 80°C for 24 h (Scheme 19Eb) (Zhang et al., 2018b). c) The Pd(OAc)_2_-catalyzed tandem reaction of *N*-(2-cyanoaryl)benzamides with arylboronic acids in 1,10-phenanthroline, TFA, and THF at 80°C for 24 h delivered 2,4-disubstituted quinazolines in 22%–97% yields (Scheme 19Ec) (Zhu et al., 2018). The important advantages of these approaches are a) bromo and iodo group/amine group/halogen and hydroxyl groups were well tolerated under the optimized conditions, which are amenable to further synthetic transformations and b) excellent chemoselectivity.

**SCHEME 20:**
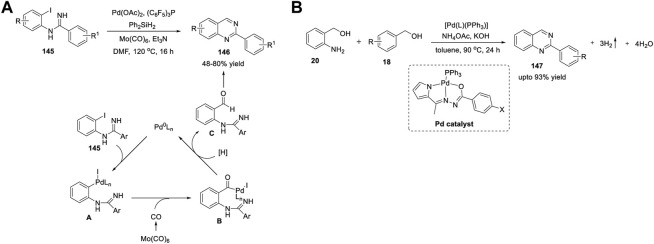
**(A)** Pd-catalyzed synthesis of quinazolines *via* reductive carbonylation **(B)** Pd-*N*,*N*,*O*-pincer type complex for the synthesis of quinazolines *via* double dehydrogenative coupling.

Similarly, Wu et al. (2021) demonstrated Pd(OAc)_2_-catalyzed reductive carbonylation of *N*-(2-iodophenyl)benzimidamide **145** using P(C_6_F_5_)_3_ as the ligand, Ph_2_SiH_2_ as the hydrogen source, Mo(CO)_6_ as the carbonyl source, and triethylamine as the base in DMF at 120°C for 16 h, providing a series of quinazolines **146** in 48%–80% yields (Scheme 20A) (Lu et al., 2021).

The considerable benefits of this protocol are that silane acts as a better nucleophile than amidine and has good functional group tolerance. An insight into the mechanistic investigation suggested that oxidative addition of Pd(0) gives arylpalladium species **A**. CO insertion followed by the generation of aldehyde intermediate **C**, aided by Ph_2_SiH_2_, is accompanied by the release of Pd(0) to close the catalytic cycle. Next, the intramolecular condensation of **C** leads to the formation of the desired quinazoline **146** (Scheme 20A).

Recently, Ramesh et al. (2022) demonstrated a new Pd(II) *N*,*N*,*O*-pincer-type catalyst for the sustainable synthesis of quinazolines *via* a sequential ADC strategy. The one-pot reaction of readily available alcohols **18** and 2-aminobenzylalcohol **20** in the presence of [Pd(L)(PPh_3_)], 4-substituted methyl-2-pyrrolyl benzhydrazone as the ligand, and ammonium acetate and KOH as the base in toluene at 90°C for 24 h produced the product **147,** with up to 93% yield (Scheme 20B). (Anandaraj et al., 2021) The major benefits of this methodology are broad substrate scope, oxidant-free, and environmental friendliness.

### 3.4 Silver

Silver, a less toxic metal, displays excellent selectivity and stability, and is more economical than other precious transition metals. Due to its Lewis acid character, Ag can act as a cocatalyst, oxidant, radical precursor, etc. Silver provides a unique platform for synthetic transformations, such as olefin and alcohol oxidation (Kolobova et al., 2019), intramolecular cyclization (Yang et al., 2019b), cycloaddition (Liu et al., 2019), and C-H activation (Ju et al., 2019).

**SCHEME 21:**
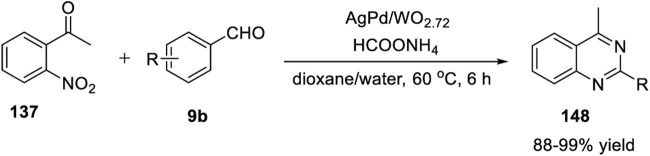
Ag/Pd NPs catalyzed synthesis of quinazolines.

Sun et al. (2017) developed Ag/Pd NPs deposited on WO_2.72_ as an efficient catalytic system for high-yield synthesis of quinazoline scaffolds. The one-pot aromatization of ammonium formate, 2-nitroacetophenone **137**, and aldehydes **9b** in the presence of the Ag_48_Pd_52_/WO_2.72_ catalyst in dioxane and water at 60°C for 6 h afforded substituted quinazolines **148** in 88%–99% yields (Scheme 21). (Yu et al., 2017) In this green protocol, the catalyst could be recycled at least five times with no loss in activity.

## 4 Third-row transition metal catalysts

### 4.1 Iridium

Iridium complexes have emerged as attractive candidates for organic catalysis because of their stability, high conversion rates, short reaction times, and excellent enantioselectivity. Iridium catalysts perform a broad array of reactions such as hydrogen transfer reactions (Anxionnat et al., 2013), photoredox reactions (Kim et al., 2020), and C-H activation (Wang et al., 2019).

**SCHEME 22:**
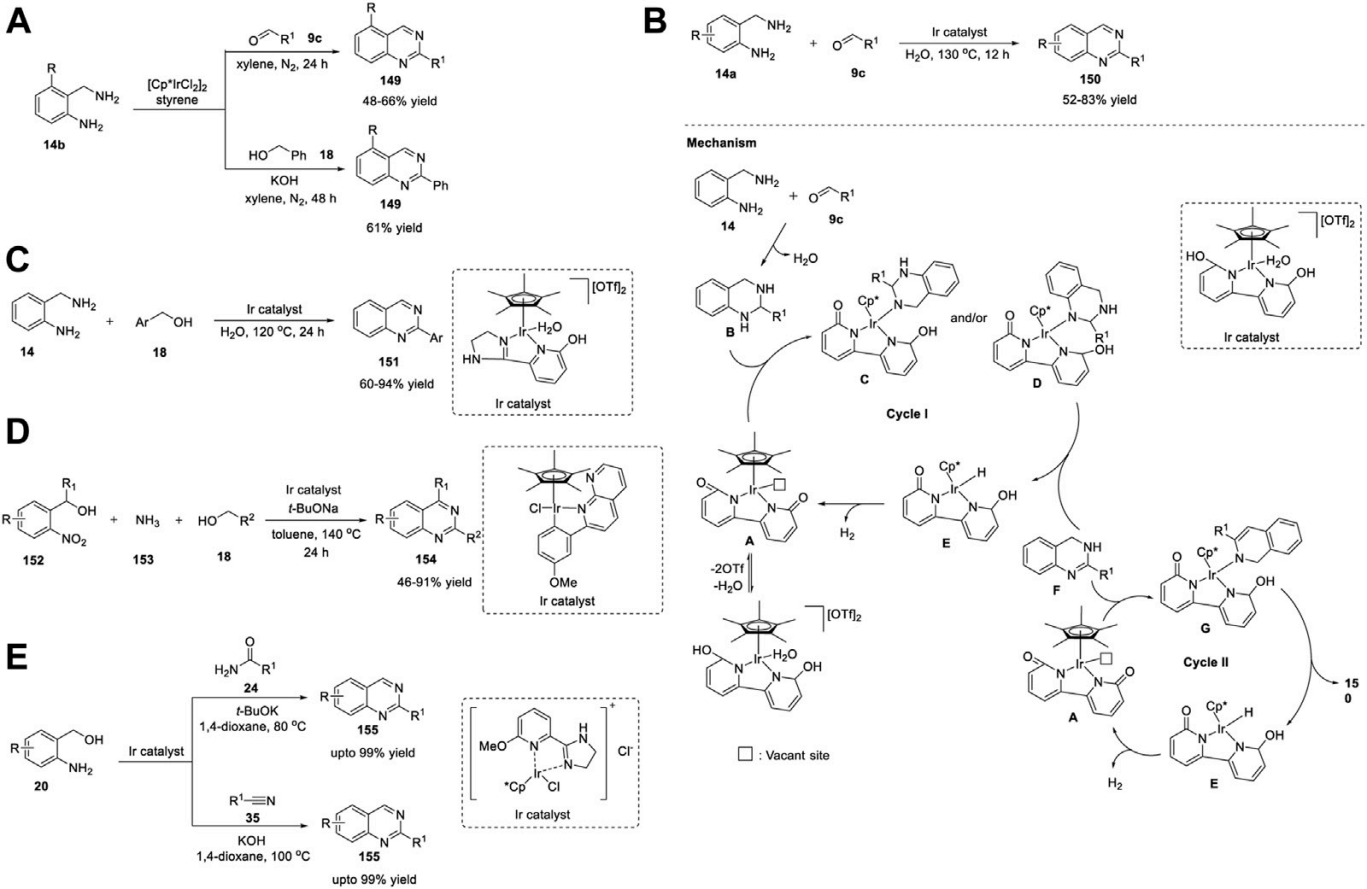
**(A)** Iridium catalyzed synthesis of quinazolines *via* hydrogen transfer strategy **(B)** Ir-catalyzed synthesis of quinazolines *via* acceptorless dehydrogenative cyclization **(C)** Ir-catalyzed synthesis of quinazolines *via* ligand promoted dehydrogenation **(D)** Naphthyridine-based Ir-catalyst for the synthesis of quinazolines **(E)** Ir-catalyzed synthesis of quinazolines *via* ADC reaction.

Zhou et al. (2013) reported a hydrogen transfer strategy for the efficient synthesis of 2-substituted quinazolines using an Ir catalyst. The one-pot reaction of 2-aminobenzylamine **14b** and benzaldehydes **9c**/benzyl alcohol **18** using [Cp*IrCl_2_]_2_ as the catalyst and styrene as the hydrogen acceptor in refluxing xylene for 24–48 h produced quinazolines **149** in 48%–66% yields (Scheme 22A). (Fang et al., 2013) Moreover, when benzyl alcohol **18** was used as the substrate, KOH was used as the base. Notably, a wide variety of aryl and alkyl aldehydes gave the desired product in good yields easily under the optimized conditions.

Similarly, Li et al. (2017) identified a novel water-soluble metal–ligand bifunctional Ir catalyst for the facile construction of quinazoline moieties *via* ADC. In the presence of the [Cp*Ir (6,6’-(OH)_2_bpy)(H_2_O)][OTf]_2_ catalyst, the ADC reaction between 2-aminobenzyl amines **14a** and benzaldehydes **9c** at 130°C for 12 h in water afforded quinazolines **150** in 52%–83% yields *via* ligand-promoted dehydrogenation (Scheme 22B). (Fan et al., 2017) Furthermore, electron-donating and electron-withdrawing groups on the aromatic ring and aliphatic aldehydes resulted in the corresponding products **150** in good yields. The attractive benefits of this environmentally benign approach include high atom efficiency and being oxidant- and organic solvent-free.

An insight into the mechanistic investigation depicted in Scheme 22B explains that the first step is the elimination of TfOH and water from the catalyst to give unsaturated species **A**. In cycle **I**, the ligand present in A accepts a proton from tetrahydroquinazoline **B**, which in turn is formed through condensation between 2-aminobenzylamine **14** and aldehyde **9c**. This results in the formation of **C** and/or **D**, which then undergo β-hydrogen elimination to form the iridium hydride species **E** and dihydroquinazoline **F**. Simultaneous transfer hydrogenation from the hydroxyl proton on the bipyridine ligand and the hydride on the iridium results in hydrogen gas liberation and regenerated catalytic species **A**. In cycle **II**, the ligand of species **A** accepts a proton of dihydroquinazoline **F** to furnish species **G**, which then undergoes β-hydrogen elimination to give iridium hydride species **E** and quinazoline **150**.

Based on the above-mentioned ligand-promoted dehydrogenation concept, Kundu et al. (2019) demonstrated a cooperative iridium complex-catalyzed synthesis of quinazolines **151** in 60%–94% yields *via* the treatment of 2-amino benzylamines **14** and alcohols **18** in water at 120°C for 24 h (Scheme 22C) (Chakrabarti et al., 2019).

In addition, 1-naphthalenemethanol and heteroatom-containing primary alcohols such as furfuryl alcohol, piperonyl alcohol, nicotinyl alcohol, and 2-thiophenemethanol, were tolerated well under these conditions and produced good yields. To date, there have been no reports of the generation of quinazolines from bio-renewable alcohols in water. The salient features of this approach are oxidant- and base-free reactions and usage of water as a green solvent.

Recently, Zhang et al. (2020) reported the first example of a novel iridium complex featuring a 2-(4-methoxyphenyl)-1,8-naphthyridyl ligand for the hydrogen-transfer-mediated annulation of 2-nitrobenzylic alcohols **152**, ammonia **153**, and alcohols **18**. The reaction proceeded using *t*-BuONa as the base in toluene at 140°C for 24 h and generated quinazolines 154 in 46%–91% yields (Scheme 22D). (Tan et al., 2020) The novelty of this transformation lies in the utilization of hydrogen for substrate activation through transfer hydrogenation (TH) of the nitro group, which in turn is generated from the dehydrogenation of alcohols and the dehydroaromatization process and hence does not require external reductants. The non-coordinated N-atom in the ligand significantly promoted the condensation step *via* hydrogen bonding. The major advantages of this method are its high step and atom efficiency, good substrate and functional group compatibility, and non-toxic byproduct such as water.

Very recently, Luo et al. (2022) reported an efficient iridium-catalyzed ADC reaction between 2-aminoarylmethanols **20** and amides **24**/nitriles **35** in the presence of *t*-BuOK/KOH in 1,4-dioxane at 80°C–100°C to provide quinazolines **155** (Scheme 22E). (Shui et al., 2022) Excellent yields of up to 99% were achieved by employing a variety of substituted 2-aminobenzyl alcohols **20**, (hetero)aryl or alkyl benzamides **24**, and nitriles **35**. The salient features of this protocol are mild reaction conditions, a simple operation procedure, and high atom economy.

### 4.2 Platinum

Platinum metals provide synthetic routes to many useful and interesting compounds. Pt catalysts have proven their versatility in several transformations such as hydrogenation (Zhang et al., 2016), hydrosilylation (Lukin et al., 2020), hydroamination (Bender and Widenhoefer, 2005), and cyclic-isomerization (Lu et al., 2009).

**SCHEME 23:**
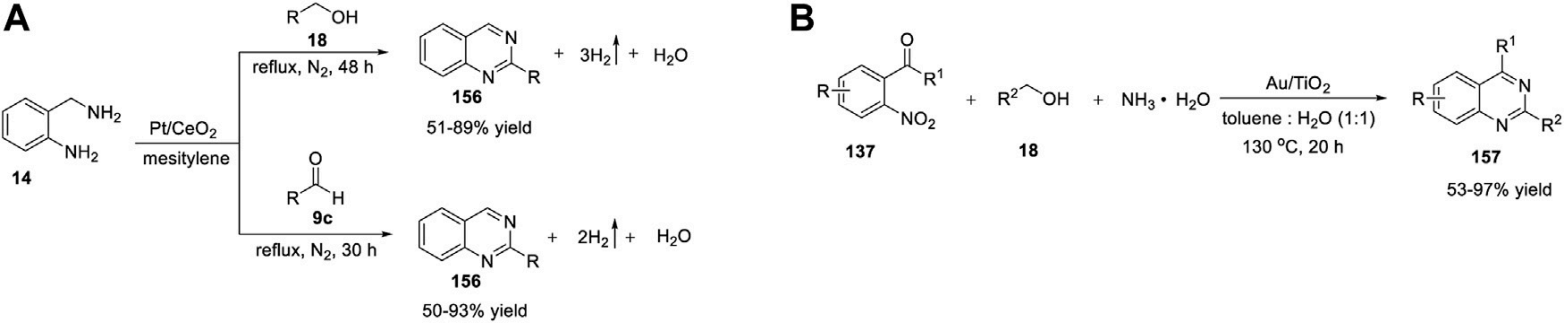
**(A)** Pt NPs catalyzed synthesis of quinazolines *via* acceptorless dehydrogenation **(B)** TiO_2_-supported Au-NPs for the synthesis of quinazolines *via* hydrogen transfer strategy.

Shimizu et al. (2014) reported the first acceptorless method for facile access to quinazolines **156** in 50%–93% yields from 2-aminobenzyl amine **14** and alcohols **18**/aldehydes **9c**, with the reaction catalyzed by a CeO_2_-supported Pt catalyst in mesitylene under reflux for 30–48 h (Scheme 23A). (Chaudhari et al., 2014) The major merits of this strategy include good catalyst reusability, low catalyst loading, wide substrate scope, and first acceptor- and additive-free catalytic systems.

### 4.3 Gold

Gold, a noble metal, possesses exclusive catalytic activities, exhibits excellent chemoselectivity, resistance to oxygen, and orthogonal reactivity, and requires mild reaction conditions. Au extends the route to a new class of homogenous and heterogeneous catalysis for a broad array of reactions such as epoxidation (Ji et al., 2018), cycloisomerization reactions (Hu et al., 2020), coupling reactions (Nijamudheen and Datta, 2019), and total syntheses of natural products (Pflasterer and Hashmi, 2016).

Wang et al. (2015) demonstrated an efficient and selective nitrogen-source-promoted hydrogen-transfer strategy for the construction of 2,4-disubstituted quinazolines **157** from *o*-nitroacetophenones **137**, alcohols **18**, and NH_3_·H_2_O (N source), with the reaction catalyzed by Au/TiO_2_ in the presence of toluene:H_2_O (1:1) at 130°C for 20 h (Scheme 23B). (Tang et al., 2015) A variety of alcohols **18** and nitro compounds **137** underwent the transformation smoothly in aqueous media, resulting in 53%–97% yields. This one-pot cascade reaction includes the dehydrogenative oxidation of alcohols and C-N bonds, reduction of the nitro group, and condensation of aldehydes with amines. The benefits of this protocol are as follows: a) no requirement of additional oxidant/additive/reducing agent, b) multiple C-N bond formation, c) good tolerance to air and water, and d) recyclable and reusable catalyst.

## 5 Conclusion

This review summarizes the methodology and importance of transition-metal catalysis in the synthesis of medicinally important quinazolines over the past decade. Reaction mechanisms are provided for every representative example, along with the advantages and limitations associated with it. Significant progress accomplished by first-row transition metals, such as Ti, Mn, Fe, Co, Ni, and Cu, is due to their high abundance, low toxicity, and low cost. Synthetic transformations performed by these metals include heterogeneous catalysis, multicomponent reactions, cyclization, one-pot or cascade or tandem reactions, C-N cross coupling, Ullmann-type condensation, aerobic oxidation, oxidative amination, C(sp^3^)–H/C(sp^2^)–H oxidative annulation, [4 + 2] cycloaddition, and annulation {[2 + 2 + 2], [3 + 2 + 1], [4 + 1 + 1]}. Despite their low availability and high cost, second- (Ru, Rh, Pd, and Ag) and third-row (Ir, Pt, and Au) transition metals catalyze unique transformations owing to their high stability and excellent selectivity. The majority of these are ADC, C-H activation ([4 + 2], aza-[4 + 2], [5 + 1]) and annulation, hydrogen transfer strategy, ligand-promoted dehydrogenative reaction, carbonylation, carbopalladation, and the use of NPs for the successful synthesis of quinazolines. In addition to homogeneous catalysis, heterogeneous catalytic approaches have also been adopted in the form of nanoporous octahedral molecular sieves, nanocomposites, ionic liquids, molecular organic frameworks, nanocubes, and magnetically recoverable nanocatalysts for the efficient synthesis of quinazolines. These catalysts tend to exhibit improved sustainability, stability, catalytic site availability, reusability, and recovery. Some of the reactions were performed using water as the green solvent and air as an oxidant, thus avoiding the use of organic solvents and excess oxidants and making the synthesis environmentally benign. New protocols with good substrate compatibility, chemoselectivity, simple operating conditions, readily available precursors, high atom economy, and high yields are always in demand. Based on the results, we believe that this review will provide a sufficient overview of transition-metal-catalyzed synthesis of quinazolines and encourage synthetic and medicinal chemists for further development in the future.
